# A mouse model of prenatal exposure to Interleukin-6 to study the developmental origin of health and disease

**DOI:** 10.1038/s41598-021-92751-6

**Published:** 2021-06-24

**Authors:** Tarak Srivastava, Trupti Joshi, Daniel P. Heruth, Mohammad H. Rezaiekhaligh, Robert E. Garola, Jianping Zhou, Varun C. Boinpelly, Mohammed Farhan Ali, Uri S. Alon, Madhulika Sharma, Gregory B. Vanden Heuvel, Pramod Mahajan, Lakshmi Priya, Yuexu Jiang, Ellen T. McCarthy, Virginia J. Savin, Ram Sharma, Mukut Sharma

**Affiliations:** 1 Section of Nephrology, Children’s Mercy Hospital and University of Missouri at Kansas City, 2401 Gillham Road, Kansas City, MO 64108 USA; 2 Midwest Veterans’ Biomedical Research Foundation (MVBRF), Kansas City, MO USA; 3 Department of Oral and Craniofacial Sciences, University of Missouri at Kansas City-School of Dentistry, Kansas City, MO USA; 4 Department of Health Management and Informatics and MU Informatics Institute, University of Missouri, Columbia, MO USA; 5 Department of Electrical Engineering and Computer Science, University of Missouri, Columbia, MO USA; 6 Christopher S. Bond Life Sciences Center, University of Missouri, Columbia, MO USA; 7 MU Data Science and Informatics Institute, University of Missouri, Columbia, MO USA; 8 Children’s Mercy Research Institute, Children’s Mercy Hospital and University of Missouri at Kansas City, Kansas City, MO USA; 9 Department of Pathology and Laboratory Medicine, Children’s Mercy Hospital and University of Missouri at Kansas City, Kansas City, MO USA; 10 Kansas City VA Medical Center, Kansas City, MO USA; 11 Department of Internal Medicine, The Jared Grantham Kidney Institute, University of Kansas Medical Center, Kansas City, KS USA; 12 Department of Biomedical Sciences, Western Michigan University Homer Stryker M.D. School of Medicine, Kalamazoo, MI USA; 13 Department of Pharmaceutical and Administrative Sciences, College of Pharmacy and Health Sciences, Drake University, Des Moines, IA USA

**Keywords:** Developmental biology, Nephrology

## Abstract

Systemic inflammation in pregnant obese women is associated with 1.5- to 2-fold increase in serum Interleukin-6 (IL-6) and newborns with lower kidney/body weight ratio but the role of IL-6 in increased susceptibility to chronic kidney (CKD) in adult progeny is not known. Since IL-6 crosses the placental barrier, we administered recombinant IL-6 (10 pg/g) to pregnant mice starting at mid-gestation yielded newborns with lower body (p < 0.001) and kidney (p < 0.001) weights. Histomorphometry indicated decreased nephrogenic zone width (p = 0.039) with increased numbers of mature glomeruli (p = 0.002) and pre-tubular aggregates (p = 0.041). Accelerated maturation in IL-6 newborns was suggested by early expression of podocyte-specific protein podocin in glomeruli, increased 5-methyl-cytosine (LC–MS analysis for CpG DNA methylation) and altered expression of certain genes of cell-cycle and apoptosis (RT-qPCR array-analysis). Western blotting showed upregulated pJAK2/pSTAT3. Thus, treating dams with IL-6 as a surrogate provides newborns to study effects of maternal systemic inflammation on future susceptibility to CKD in adulthood.

## Introduction

The increasing global prevalence of chronic kidney disease (CKD) and obesity is associated with chronic inflammation identified by elevated pro-inflammatory cytokines including serum interleukin 6 (IL-6)^1–4^. Recent epidemiological studies have shown that maternal overweight/obesity is associated with low kidney volume/birth weight ratio, increased incidence of CKD, and increased risk for congenital anomalies in their children^5–11^. These observations suggest that maternal obesity (gestational inflammation) alters the intrauterine environment affecting kidney development (fetal programming), which may predispose the offspring to increased susceptibility to CKD in adulthood.

The developmental origin of health and disease hypothesis (or Barker’s hypothesis) suggests that perinatal events influence the genesis of a disease, such as CKD in adulthood^12^. In the framework of a ‘two-hit’ model, changes in fetal kidney programming becomes the ‘first hit’, and any subsequent injury in the form of loss of one kidney due to trauma or tumor, obesity, malnutrition, etc. results in the ‘second hit’. Thus, the changes induced by the ‘first hit’ influence the course and outcome of kidney disease from the ‘second hit’^13,14^. Lack of a convenient animal model is one of the reasons for our limited understanding on the effects of maternal systemic inflammation in causing impairment of kidney development and long-term susceptibility to CKD in the progeny. Hence, we explored the use of prenatal treatment of pregnant mice to a pro-inflammatory cytokine (IL-6) as a surrogate of maternal (gestational) inflammation.

The placental barrier separates the maternal and fetal compartments, and regulates transfer of specific maternal proteins (e.g., certain immunoglobulins) to the fetal compartment^15^. Others have shown that, among the inflammatory cytokines associated with obesity (IL-6, TNFα, IL-1β), only maternal IL-6 crosses the placental barrier starting in mid-gestation^16–18^. Since serum IL-6 is elevated 1.5–2-fold in pregnant obese women compared to non-obese pregnant women, we postulated that transfer of increased maternal IL-6 may alter kidney fetal programming and initiate the ‘first hit’ in a chain of pro-inflammatory changes^19–21^.

Here we show that prenatal administration of IL-6 to pregnant mice resulted in offspring with decreased birth weight, decreased kidney weight, and accelerated maturation of the developing kidney. Since CKD is associated with glomerular dysfunction resulting in albuminuria/proteinuria^22,23^, we tested the direct effect of IL-6 on the glomerular filtration barrier function. We found that incubation with IL-6 resulted in derangement of cultured podocyte cytoskeleton and increased glomerular albumin permeability in vitro.

Our approach permits controlled dosing of IL-6 starting in mid-gestation, yields sufficient number of newborns for studies and permits clear interpretation of results for establishing specific causal relationship between maternal systemic inflammation and fetal kidney development. We propose that newborns from mothers treated with IL-6 will be a convenient mouse model to study the long-term effects of maternal systemic inflammation during pregnancy and investigate susceptibility to CKD following a second injury in adult progeny in future studies.

## Results

We evaluated the effect of intraperitoneal injection of IL-6 to pregnant mice starting in mid-gestation to study its in vivo effect on intrauterine kidney development and performed in vitro experiments to determine whether IL-6 directly (1) impairs the glomerular filtration barrier function, (2) affects the podocyte actin cytoskeleton, changes that precede overt proteinuria and, (3) hampers metanephric kidney growth in culture. Results outlined below suggest that IL-6 has direct effect on fetal kidney development and on glomerular filtration barrier. These effects of IL-6 during kidney development may have long-term impact and increase the susceptibility to CKD in the adult offspring.

### Interleukin-6 administration to pregnant dam had no detrimental effect on the mother

Intraperitoneal (IP) administration of IL-6 at 10 pg/g BWT, every other day from E12.5 to end of gestation (E20 ± 0.5) to timed-pregnant C57BL/6 mice had no detrimental effect on the mother. There were no miscarriages or deaths in pregnant dams. We did not observe decreased food/water intake, decreased activity or decreased grooming in the IL-6 injected pregnant dams.

### IL-6 administration to pregnant mice resulted in smaller newborns and smaller kidneys

**Figure 1:**
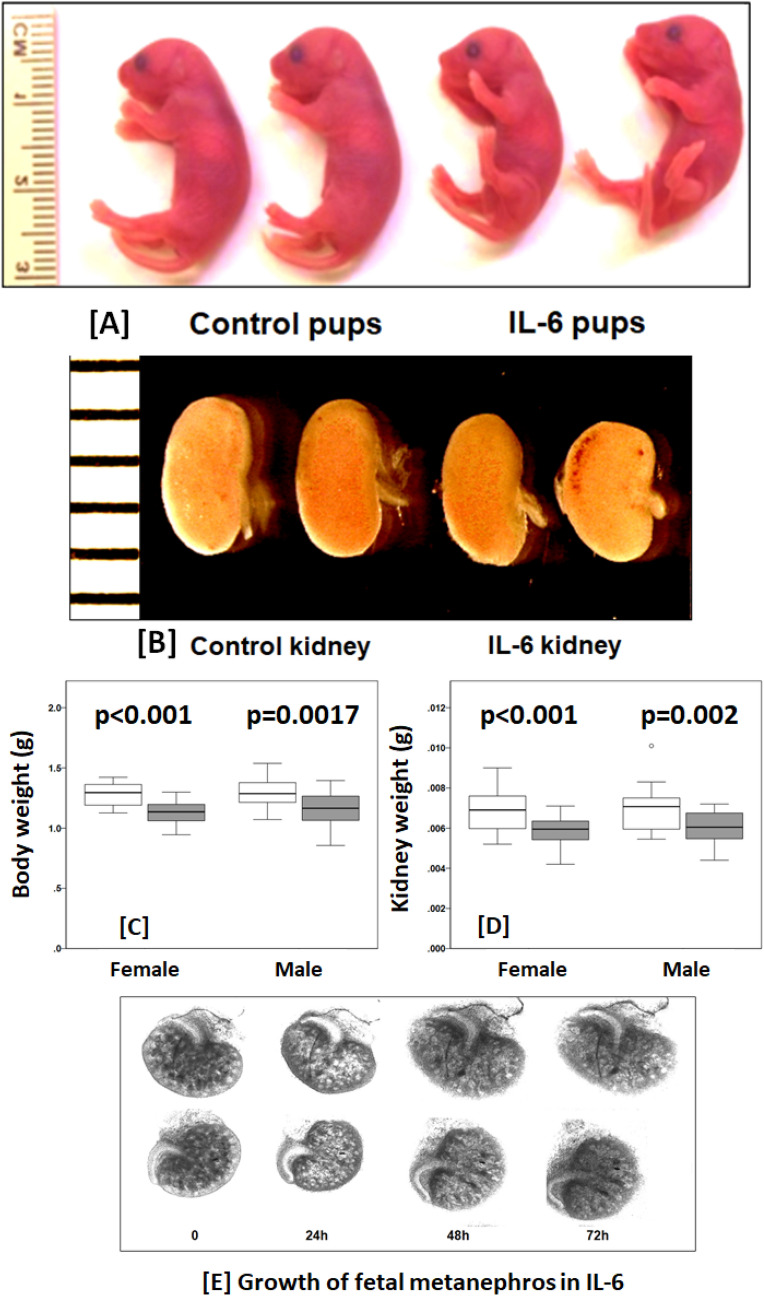
IL-6 treatment alters kidney growth in vivo and in vitro. (**A**) Representative images newborns and (**B**) newborn kidneys from control dams and those treated with IL-6 (10 pg/g BWT, ip) every other day (E12.5–E20). Maternal IL-6 treatment resulted in smaller body size and kidneys in the newborn compared to pups born to control dams. Box plots show the distribution of (**C**) body weights and (**D**) kidneys by sex. Body and kidney weights in both sexes were significantly different between control (n = 53) and IL-6 groups (n = 48) (see text for details). (**E**) Mouse fetal metanephroi isolated at E13.5 were grown in vitro with 10 pg/mL (IL-6) or without (Control) for 0, 24, 48 and 72 h. IL-6 caused decrease in the growth of metanephric kidneys determined by reduction in surface area.

Figure 1A–D shows the results of mid-gestation intraperitoneal (IP) administration of IL-6 (10 pg/g BWT, every other day from E12.5 to end of gestation (E20 ± 0.5) to timed-pregnant C57BL/6 mice. A total of 53 pups (27 female and 26 male) were born to control dams (n = 6) and 48 pups (24 female and 24 male) to IL-6 treated dams (n = 6). IL-6 treatment did not result in mortality or affect the number of male or female newborns (Supplementary Table S1). The pups born to IL-6 treated dams appeared normal except being smaller compared to pups born to control dams (Fig. 1A). The mean ± SD birth weight was lower in pups born to IL-6 treated dams (1.144 g ± 0.121, n = 48) compared to control dams (1.282 g ± 0.111, n = 53; p < 0.001). The ratio of body weight between the Control and IL-6 groups was 1:0.89.

Figure 1C shows the box-plot distribution of newborn body weight by sex. The difference between the Control and IL-6 groups for body weight for female pups was 1.277 g ± 0.093, n = 27 *vs*. 1.129 g ± 0.093, n = 24, p < 0.001 and male pups was 1.286 g ± 0.128, n = 26 *vs*. 1.158 g ± 0.145, n = 24, p = 0.0017. Within each group, body weights of male and female (male:female) pups were comparable i.e., Control group (1:0.99, p = 0.77) and IL-6 group (1:0.98, p = 0.43).

Every newborn had two kidneys, but the kidneys were smaller in pups born to IL-6 treated dams as compared to the pups born to control dams (Fig. 1B). The kidney weight (mean ± SD) was lower in pups born to IL-6 treated dams (0.0059 g ± 0.0009, n = 48) compared to the control group (0.0069 g ± 0.0011, n = 53; p < 0.001). The kidney weight ratio between the Control and IL-6 groups was 1:0.86 (Control:IL-6). Similar observation was made regarding weights of left and right kidneys. Thus, the left and right kidney weight ratios were 1:0.89 (Control:IL-6 groups) and 1:0.84 (Control:IL-6 groups), respectively.

Figure 1D shows the box-plot distribution of mean kidney weight by sex. The difference between the Control and IL-6 groups for kidney weight of female pups was significant at p < 0.001 (0.0069 g ± 0.0010, n = 27 *vs*. 0.0058 g ± 0.0009, n = 24) and that of male pups was p = 0.002 (0.0070 g ± 0.0011, n = 26 *vs*. 0.0061 g ± 0.0007, n = 24). Within each respective group, there was no sex difference in fetal kidney weight between male pups and female pups within the control group (1:0.99, p = 0.86) and IL-6 group (1:0.96, p = 0.32).

The data was also analyzed using all newborns from each dam as a single dataset point. The results were essentially unchanged. The mean ± SD birth weight was lower in pups born to IL-6 treated dams (1.138 g ± 0.102, n = 6) compared to control dams (1.276 g ± 0.078, n = 6; p = 0.025). The ratio of body weight between the Control and IL-6 groups was 1:0.89. The difference between the Control and IL-6 groups for body weight of female pups was 1.263 g ± 0.070, n = 6 *vs*. 1.124 g ± 0.077, n = 6, p = 0.008 and that of male pups was 1.295 g ± 0.095, n = 6 *vs*. 1.161 g ± 0.133, n = 6, p = 0.074. The kidney weight (mean ± SD) was lower in pups born to IL-6 treated dams (0.0060 g ± 0.0004, n = 6) compared to the control group (0.0069 g ± 0.0008, n = 6; p = 0.037). The kidney weight ratio between the Control and IL-6 groups was 1:0.87 (Control:IL-6).The difference between the Control and IL-6 groups for body weight of female pups was 0.0068 g ± 0.0007, n = 6 *vs*. 0.0059 g ± 0.0003, n = 6, p = 0.024 and male pups was 0.0070 g ± 0.0009, n = 6 *vs*. 0.0061 g ± 0.0005, n = 6), p = 0.073. The boxplot distribution is shown as Supplementary Figure S1.

The kidney weight/body weight ratio was lower but not significant in pups born to IL-6 treated dams (0.0052 ± 0.0006, n = 48) compared to the control group (0.0054 ± 0.0006, n = 53; p = 0.09). The kidney weight/body weight ratio in pups born to IL-6 treated dams (0.0052 ± 0.0002, n = 6) was similar to the control group (0.0053 ± 0.0004, n = 6; p = 0.61).

Figure 1E shows the growth of metanephroi (E13.5) incubated with IL-6 (10 pg/mL) for up to 72 h. Morphometric analysis using NIH Image J software for calculating surface area at 0, 24, 48 and 72 h showed that the surface area of the Control group increased by 37% and that of the IL-6 group by 30%. Tissue growth ratio between the Control and IL-6 groups was 1:0.81. Thus, IL-6 attenuated the growth of developing metanephroi in vitro and fetal kidney in vivo by ~ 15–20%.

### Gestational IL-6 administration to dams resulted in upregulation of JAK-STAT pathway in newborn kidneys

**Figure 2:**
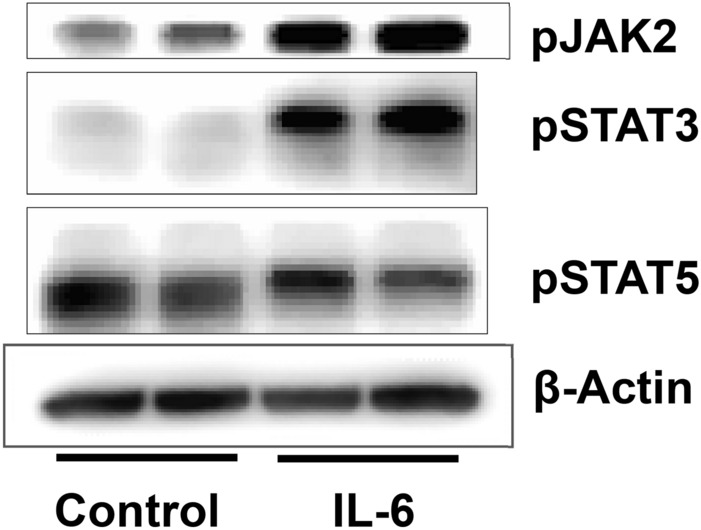
Gestational administration of IL-6 to dams results in upregulation of JAK/STAT pathway in newborn kidneys. Western blot analysis showing changes in phosphorylation of JAK2, STAT3 and STAT5 in kidneys of pups born to dams treated with saline (Control) versus IL-6. Kidneys all newborns from each dam (n = 2, kidneys from 6 to 8 pups included in each sample) were pooled to obtain protein lysate for the Western blot. Increased phosphorylation of JAK2 and STAT3 but not STAT5 was observed. β-Actin was used as the loading control.

Newborn kidneys were pooled to obtain protein lysates for Western blot analysis. Each lane represents pooled newborn kidneys from one dam in Fig. 2. Results show that injection of IL-6 to pregnant mice resulted in increased phosphorylation of JAK2 (Tyr1007) and STAT3 (Tyr705) in the newborn kidneys. These results corroborate the data obtained from similar experiments using podocyte cultures (vide infra*,* Fig. 9). Interestingly, IL-6 has opposite effects on pSTAT3 and pSTAT5 phosphorylation in kidneys at birth underscoring differences in STAT isoforms. These results show a definitive upregulated JAK-STAT signaling in developing kidneys caused by IL-6 injected to mothers.

### Newborn kidney morphology suggests accelerated maturation following IL-6 exposure during gestational development

**Figure 3:**
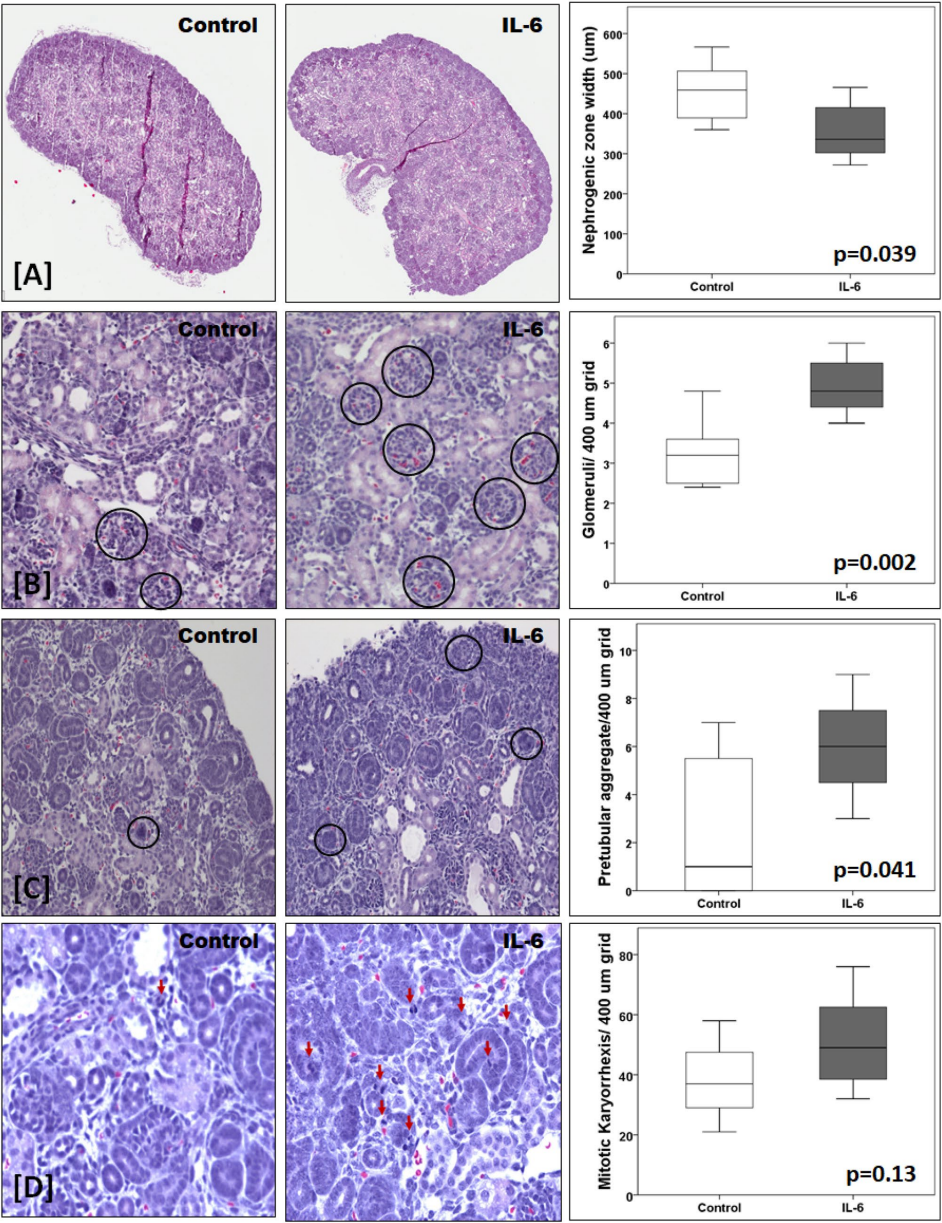
IL-6 injection to pregnant mice induces morphological changes in newborn kidneys. (**A**) Representative image of newborn kidney from control dam treated with normal saline (left) and newborn kidney from dam treated with IL-6 (10 pg/g intraperitoneally) starting in mid-gestation (middle). The box-plot distribution of nephrogenic zone width is shown in the upper right panel (n = 7 in each group). The nephrogenic zone width was significantly higher in control pups (p = 0.039). The second panel (**B**), third panel (**C**) and lower panel (**D**) each shows a representative kidney tissue of a pup born to control dam (left) and to dam treated with IL-6 (middle). The box-plot distribution of number of mature glomeruli, pretubular aggregate and mitotic karyorrhexis measured per grid in a 400 µm × 400 µm grid over 5 separate areas is shown in the right panels (n = 7 in each group). The number of mature glomeruli per grid (**B**) was significantly lower in control pups (p = 0.002). The number of pretubular aggregates per grid was significantly lower in control pups (p = 0.041). The number of cells showing mitotic karyorrhexis per grid was lower in control pups (p = 0.13).

Figure 3A–D captures the results of various morphological parameters of the newborn kidneys to highlight the effect of maternal IL-6. Figure 3A shows that the nephrogenic zone width (mean ± SD of five randomly selected cortical regions in each sample) in newborn kidneys from dams treated with IL-6 was 358.4 ± 74.3 μm (n = 7) and significantly lower compared to control pups from mothers treated with saline (453.9 ± 80.5 μm, n = 7; p = 0.039). Mature glomeruli and pre-tubular aggregates were counted over 5 separate regions using a 400 µm × 400 µm area grid. Figure 3B shows that the number of mature glomeruli per grid in kidneys from pups exposed to maternal IL-6 (4.94 ± 0.76/grid, n = 7) was significantly higher compared to fetal kidneys from control pups born to dams given saline (3.23 ± 0.87/grid, n = 7, p = 0.002). Likewise, maternal IL-6 also influenced the number of pre-tubular aggregates. Figure 3C shows that the number of pre-tubular aggregate/grid in kidneys from pups exposed to maternal IL-6 (6.00 ± 2.16/grid, n = 7) was significantly higher compared to kidneys from control pups (2.71 ± 3.15/grid, n = 7, p = 0.041). Figure 3D shows results of further histological evaluation that revealed increased mitotic karyorrhexis (a cell in mitosis while also undergoing the process of karyorrhexis) in pups gestationally exposed to increased maternal IL-6. The mean mitotic karyorrhexis value was higher in pups exposed to IL-6 (51.29 ± 16.29 per grid, n = 7) compared to control pups (38.43 ± 13.59 per grid, n = 7, p = 0.13).

**Figure 4:**
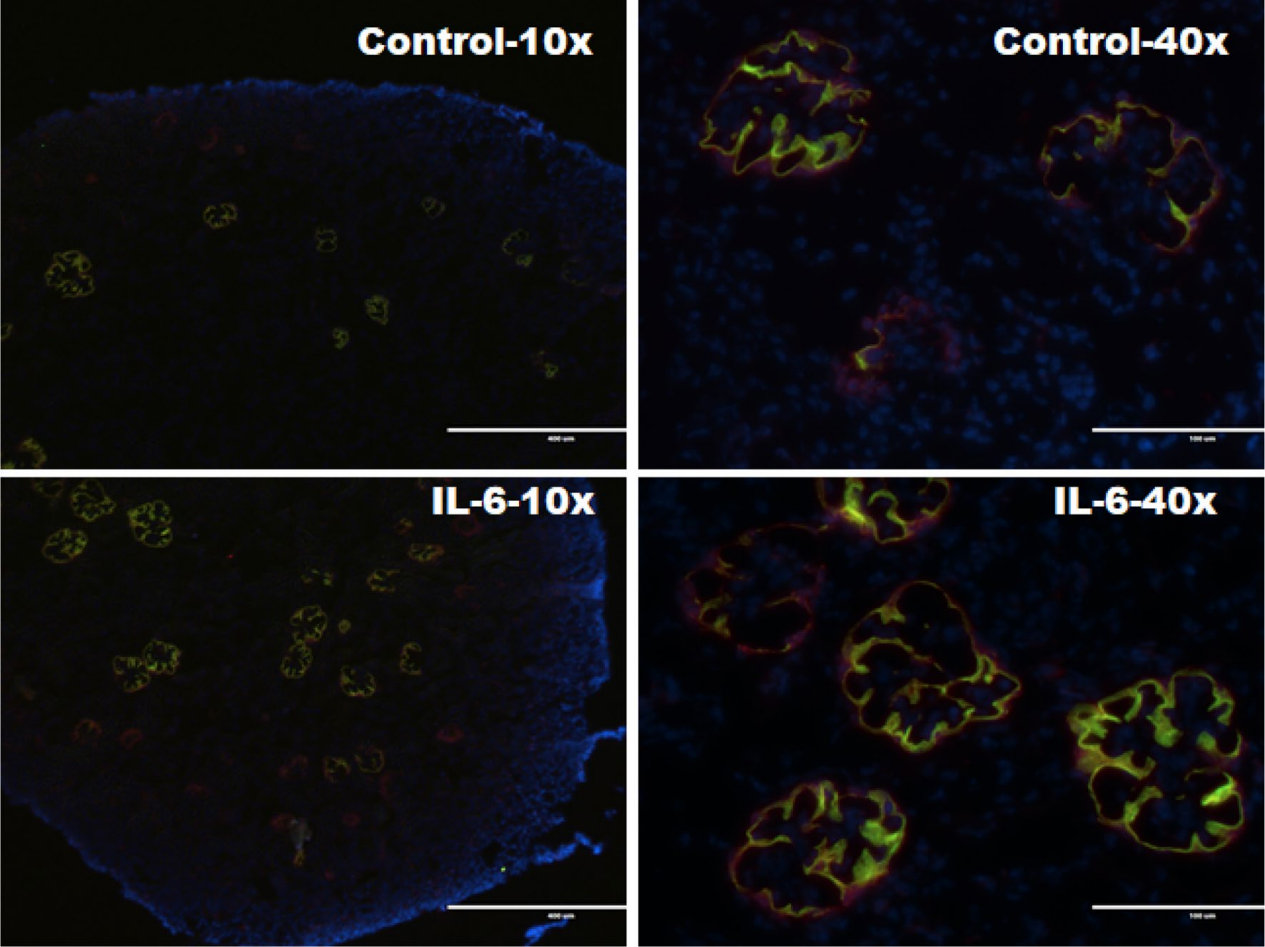
Double immunofluorescence staining with podocyte proteins podocalyxin and podocin showed increased number of mature glomeruli in newborn kidneys. Immunofluorescence microscopy was performed after double immuno-staining for podocyte proteins podocin (green) and podocalyxin (red). Representative glomerular images (left panel at ×100 and right panel at ×400 magnifications) from newborn kidneys of saline-treated dams (Control) or IL-6 treated dams (10 pg/g, IP) starting in mid-gestation.

Additionally, accelerated maturation at cellular level was evident from double immunostaining for podocytes proteins podocalyxin and podocin as described previously to identify mature glomeruli^24^. Podocytes are essential for glomerular structure–function integrity and during development express podocalyxin early but podocin later. Analysis for the number of mature glomeruli per 100× field following immunostaining for podocin and podocalyxin showed that the number of glomeruli expressing both podocalyxin and podocin was significantly higher in kidneys from pups born to dams treated with IL-6 (25.30 ± 3.95/field, n = 2) compared to kidneys from control pups born to dams given saline (11.92 ± 3.28/field, n = 2) (Fig. 4).

Overall, morphological examination from various aspects suggests that increased IL-6 accelerates kidney development resulting in small kidneys under environmental stress of maternal inflammation that can have lasting effects on kidney function in the offspring.

### Cell cycle and apoptosis gene expression was altered in kidneys of newborns of mothers treated with IL-6 during gestation

**Figure 5:**
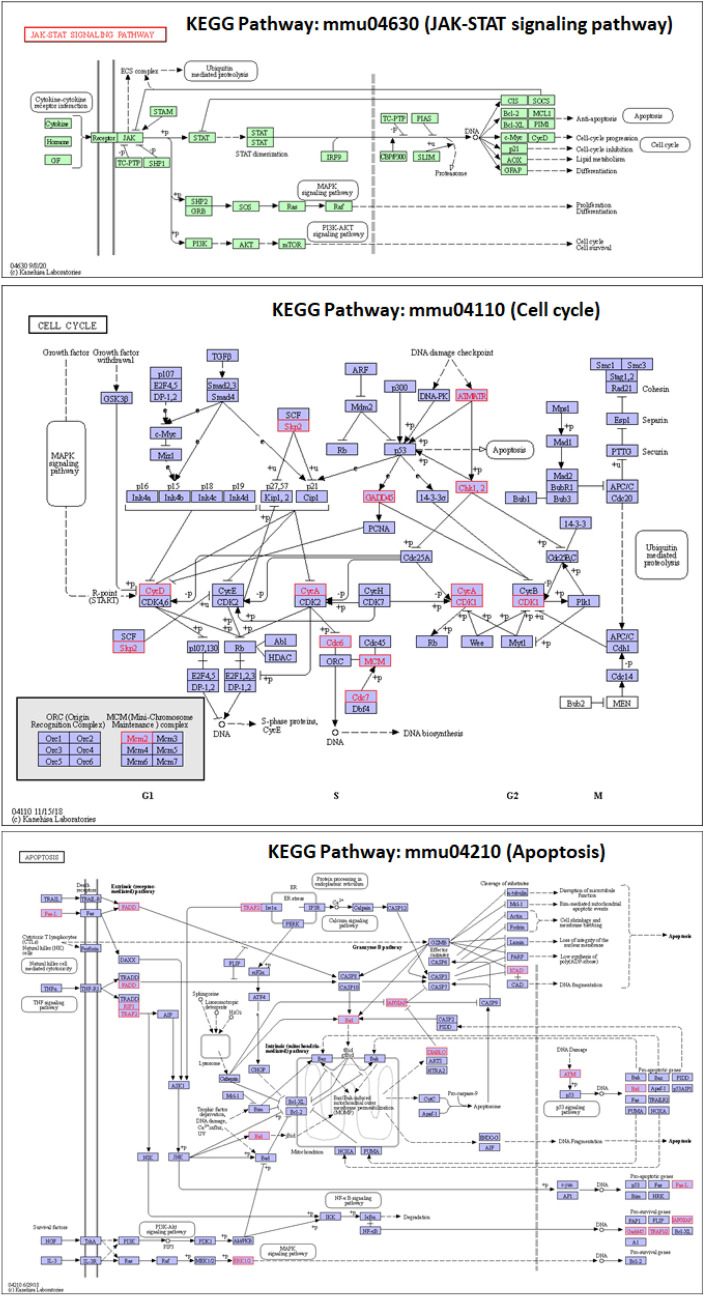
JAK-STAT, cell cycle and apoptosis pathways (KEGG pathways). JAK-STAT pathway plays a role in cell cycle and apoptosis. Genes with significantly different expression were identified using specific RT^2^ Profiler PCR Arrays for cell cycle and apoptosis. Of these, 14 (11.4% of 123 genes) of cell cycle gens and 11 (8.1% of 136 genes) of apoptosis genes were mapped to KEGG pathways (adapted with permission from Kanehisa Laboratories (KEGG Database Project)^88,89^). Mapped genes are highlighted in red font.

The observed changes outlined above including accelerated maturation, increased pre-tubular aggregates, and increased mitotic karyorrhexis led us to investigate the changes in gene expression in cell cycle and apoptosis pathways in this model. Newborn kidneys from each dam were pooled to obtain mRNA and 5–6 replicates for cell cycle and apoptosis were analyzed. Results of 96-well plate RT^2^ Profiler PCR Arrays shown in Tables 1 and 2, and Supplementary Tables 2 and 3 include the fold-change and p-values with brief description of their roles in mouse cell cycle pathway and mouse apoptosis pathway. Figure 5 shows how these differentially expressed genes are matched on the KEGG pathways. The Cell Cycle PCR Array analysis showed that 47 (56.0%) of the 84 genes on the array plate were annotated in 123 genes in the KEGG Cell Cycle (ko04110) pathway. Of the 19 genes differentially expressed in the PCR array plate, 14 genes were mapped to the KEGG pathway. Thus, 29.8% (14/47) or 11.4% (14/123) genes were differentially expressed in the KEGG Pathway. Results of the Apoptosis PCR Array analysis showed that 41 (48.8%) of the 84 genes on the array plate were annotated in 136 genes in the KEGG Apoptosis (ko04210) pathway. Of the 18 genes differentially expressed in the PCR array, 11 were mapped to the KEGG pathway. Thus, 26.8% (11/41) or 8.1% (11/136) genes were differentially expressed in the KEGG Pathway. While validation of genes of cell cycle and apoptosis is a part of our future plans, these results are consistent with the interpretation that altered gene expression in these two pathways drives the observed accelerated maturation induced by elevated levels of IL-6 during gestation (Tables 1, 2, Supplementary Tables 2, 3).

**Table 1:** The genes with their role in phases of cell cycle and regulation of cell cycle, fold change (FC) and p value performed on Qiagen’s RT^2^ Profiler PCR array gene expression analysis (mouse cell cycle panel PAMM-020ZA) in kidneys from newborn exposed to increased maternal Interleukin-6 compared to control.

Gene	Description	FC	p value	G1 phase and G1/S transition	S phase and DNA replication	G2 phase and G2/M transition	M phase	Cell cycle checkpoint and cell cycle arrest	Regulation of the cell cycle	Negative regulation of cell cycle
**Atm**	**Ataxia telangiectasia mutated homolog**	**2.29**	**0.000073**							**Negative Regulation of Cell Cycle**
**Ccna1**	**Cyclin A1**	**1.93**	**0.000033**				**M Phase**		**Regulation of the Cell Cycle**	
**Cdc7**	**Cell division cycle 7**	**1.78**	**0.002318**		**S phase and DNA Replication**					
**Chek1**	**Checkpoint kinase 1 homolog**	**1.77**	**0.00677**			**G2 Phase and G2/M Transition**		**Cell Cycle Checkpoint and Cell Cycle Arrest**		
**Trp63**	**Transformation related protein 63**	**1.73**	**0.000648**							**Negative Regulation of Cell Cycle**
**Skp2**	**S-phase kinase-associated protein 2 (p45)**	**1.59**	**0.001636**	**G1 Phase and G1/S Transition**					**Regulation of the Cell Cycle**	
**Cdk5rap1**	**CDK5 regulatory subunit associated protein 1**	**1.59**	**0.03108**					**Cell Cycle Checkpoint and Cell Cycle Arrest**		
**Chek2**	**CHK2 checkpoint homolog**	**1.54**	**0.004929**					**Cell Cycle Checkpoint and Cell Cycle Arrest**		
**Cdc6**	**Cell division cycle 6 homolog**	**1.51**	**0.001708**		**S phase and DNA Replication**		**M Phase**		**Regulation of the Cell Cycle**	
*Atr*	*Ataxia telangiectasia and rad3 related*	*1.46*	*0.000461*					*Cell Cycle Checkpoint and Cell Cycle Arrest*	*Regulation of the Cell Cycle*	
*Ccna2*	*Cyclin A2*	*1.46*	*0.033909*						*Regulation of the Cell Cycle*	
*Ccnd2*	*Cyclin D2*	*1.42*	*0.000871*						*Regulation of the Cell Cycle*	
***Aurka***	***Aurora kinase A***	***1.4***	***0.012372***						***Regulation of the Cell Cycle***	
***Cdk1***	***Cyclin-dependent kinase 1***	***1.19***	***0.012966***				***M Phase***	***Cell Cycle Checkpoint and Cell Cycle Arrest***	***Regulation of the Cell Cycle***	
***Mki67***	***Antigen identified by monoclonal antibody Ki 67***	***1.21***	***0.016439***		***S phase and DNA Replication***					
***Ccnd3***	***Cyclin D3***	***1.17***	***0.017355***						***Regulation of the Cell Cycle***	
***Mcm2***	***Minichromosome maintenance deficient 2 mitotin***	***1.34***	***0.017567***		***S phase and DNA Replication***					
***Notch2***	***Notch gene homolog 2***	***1.36***	***0.024446***					***Cell Cycle Checkpoint and Cell Cycle Arrest***		
***Gadd45a***	***Growth arrest and DNA-damage-inducible 45 alpha***	***− 1.25***	***0.028246***					***Cell Cycle Checkpoint and Cell Cycle Arrest***	***Regulation of the Cell Cycle***	

The genes with FC > 1.5 and p value < 0.05 are shown in bold, FC > 1.4 and p value < 0.05 are shown in italics and genes with FC ≤ 1.4 and p value < 0.05 are shown in bold italics. The role of these genes in different phases of cell cycle are also presented.

**Table 2:** The genes with their role in apoptosis, fold change (FC) and p value performed on Qiagen’s RT^2^ Profiler PCR Array Gene Expression Analysis (Mouse Apoptosis Panel PAMM-012ZA) in kidneys from newborns exposed to increased maternal Interleukin-6 compared to control dams.

Gene	Description	FC	p value	Induction of apoptosis	Anti-apoptotic	Death domain proteins/DNA damage	Caspases	Positive regulation	Negative regulation
**Tnfrsf11b**	**Tumor necrosis factor receptor superfamily, member 11b (osteoprotegerin)**	**2.03**	**0.000013**			**Death Domain Proteins**			
**Trp63**	**Transformation related protein 63**	**1.63**	**0.001611**	**Induction of Apoptosis**	**Anti-Apoptotic**	**DNA Damage**			
**Traf1**	**Tnf receptor-associated factor 1**	**1.56**	**0.000064**					**Positive Regulation**	
**Atf5**	**Activating transcription factor 5**	**1.54**	**0.000052**		**Anti-Apoptotic**				
**Il10**	**Interleukin 10**	**− 1.76**	**0.003926**		**Anti-Apoptotic**				
*Fasl*	*Fas ligand (TNF superfamily, member 6)*	*1.47*	*0.006512*	*Induction of Apoptosis*				*Positive Regulation*	
***Bcl2l10***	***Bcl2-like 10***	***− 1.38***	***0.000557***		***Anti-Apoptotic***		***Caspase Activators***		***Negative Regulation***
***Xiap***	***X-linked inhibitor of apoptosis***	***1.4***	***0.00135***		***Anti-Apoptotic***		***Caspase Inhibitors***		***Negative Regulation***
***Traf2***	***Tnf receptor-associated factor 2***	***1.22***	***0.004307***					***Positive Regulation***	
***Pycard***	***PYD and CARD domain containing***	***1.35***	***0.004527***	***Induction of Apoptosis***			***Caspases/Caspase Activators***	***Positive Regulation***	
***Casp4***	***Caspase 4, apoptosis-related cysteine peptidase***	***− 1.27***	***0.006693***	***Induction of Apoptosis***			***Caspases***	***Positive Regulation***	
***Dffa***	***DNA fragmentation factor, alpha subunit***	***1.26***	***0.016719***	***Induction of Apoptosis***					***Negative Regulation***
***Fadd***	***Fas (TNFRSF6)-associated *via* death domain***	***1.27***	***0.024924***	***Induction of Apoptosis***		***Death Domain Proteins***		***Positive Regulation***	
***Bid***	***BH3 interacting domain death agonist***	***1.32***	***0.026291***	***Induction of Apoptosis***				***Positive Regulation***	
***Diablo***	***Diablo homolog (Drosophila)***	***1.15***	***0.026748***	***Induction of Apoptosis***					
***Gadd45a***	***Growth arrest and DNA-damage-inducible 45 alpha***	***− 1.22***	***0.027658***					***Positive Regulation***	
***Ripk1***	***Receptor (TNFRSF)-interacting serine-threonine kinase 1***	***1.21***	***0.039765***			***Death Domain Proteins***			
***Mapk1***	***Mitogen-activated protein kinase 1***	***1.16***	***0.0467***	***Induction of Apoptosis***					

The genes with FC > 1.5 and p value  < 0.05 are shown in bold, FC > 1.4 and p value < 0.05 are shown in italics and genes with FC ≤ 1.4 and p value  < 0.05 are shown in bold italics. The role of these genes in apoptosis are also presented.

### Gestational treatment of dams with IL-6 resulted in increased total genomic DNA methylation in the newborn kidney

Elevated levels of cytosine methylation correlate with more differentiated state^25^. Newborn kidneys from each dam were pooled to obtain genomic DNA. IL-6 administration to mothers during gestation increased 5-methyldeoxycytosine (5mdC) in the newborn kidney (4.15 ± 0.046, n = 3) compared to control (3.78 ± 0.057, n = 3, p < 0.05). Levels of 5-hydroxymethyldeoxycytosine (5hmdC) in the IL-6 exposed fetal kidney (0.124 ± 0.013, n = 3) remained unchanged compared to control (0.125 ± 0.008, n = 3, p = NS). An approximately 10% increase in methylated cytosine, but not hydroxymethylated cytosine, was detected in pups born to IL-6 treated mothers.

Thus, gross and tissue morphometric analyses of glomerular and tubular development, immunofluorescence analysis of podocyte specific protein, gene expression analysis for cell cycle and apoptosis, and mass spectrometric analysis of methylated cytosine in DNA strengthen the idea that elevated maternal IL-6 induces a more differentiated state of the newborn kidney. These findings constitute the foundation of our plans to study newborns from IL-6 treated mothers for increased susceptibility to kidney disease during adulthood.

### Interleukin-6 increased glomerular albumin permeability (P_alb_) in vitro

**Figure 6:**
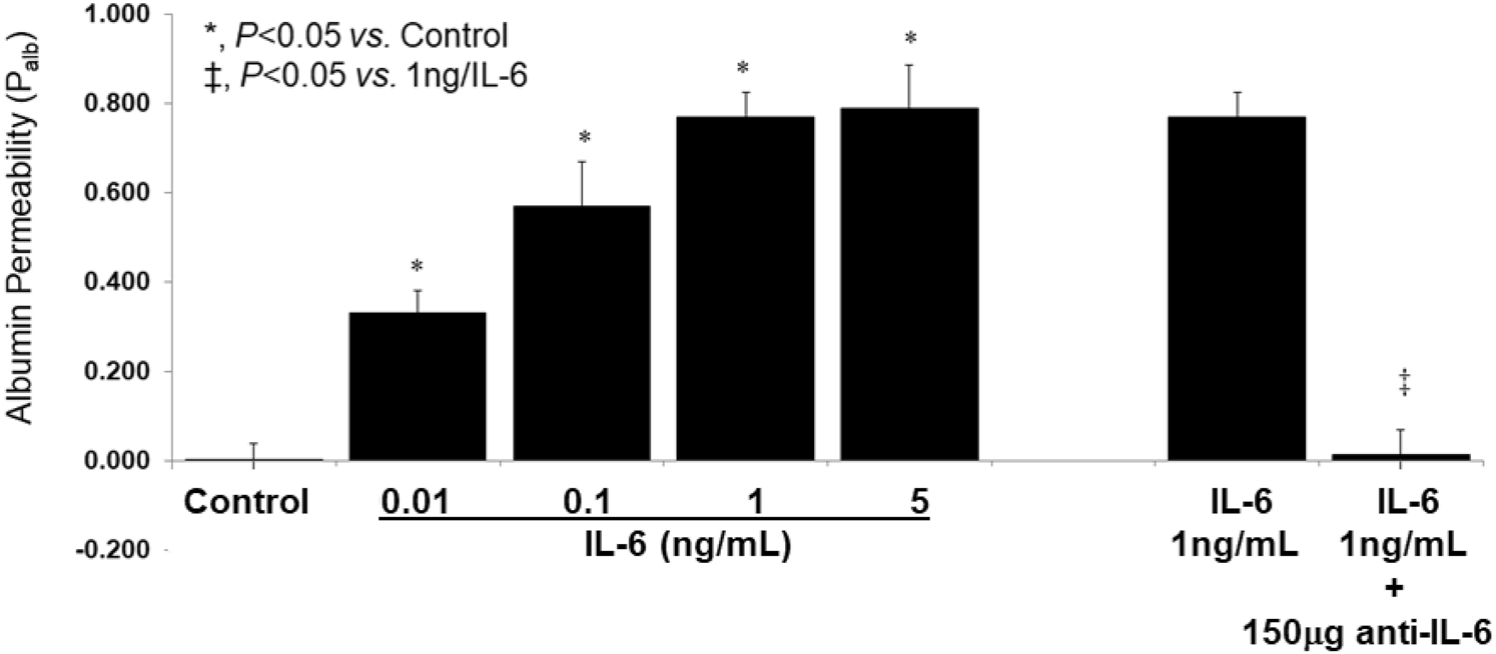
IL-6 increases glomerular albumin permeability (P_alb_) in vitro. Isolated rat glomeruli were incubated with IL-6 alone or after pretreatment with anti-IL-6 antibody for 15 min at 37 °C followed by video microscopy, determination of glomerular volume change and calculation of P_alb_. (**A**) IL-6 (0.01–5 ng/mL) caused significant dose-dependent increase in P_alb_ at 0.1–1 ng/mL (*p < 0.05 *vs*. control) with no further increase in IL-6 between 1 and 5 ng/mL. (**B**) Pretreatment of glomeruli with anti-IL-6 antibody (150 µg/mL) followed by addition of IL-6 (1 ng/mL) blocked the effect of IL-6 (*p < 0.05 *vs.* IL-6 1 ng/mL). Values are means ± SE of P_alb_ determinations in 20 glomeruli from 4 rats (5 glomeruli/rat n = 4 rats).

Previously, we have shown that increased P_alb_ indicates injury to the glomerular filtration barrier which precedes the onset of overt proteinuria in a spectrum of kidney diseases^26–29^. Present results demonstrate a direct effect of IL-6 (0.01–5 ng/mL) on P_alb_ within 15 min of incubation. Figure 6 shows that IL-6 increased P_alb_ in a dose-dependent manner. A significant increase in P_alb_ was noted at IL-6 concentration of 0.01 ng/mL, with maximal effect at 1 ng/mL (p < 0.05). Pre-treatment with anti-IL-6 antibody completely blocked the increase in P_alb_ using 1 ng/mL IL-6 (p < 0.05). These findings indicate a role for IL-6 in the glomerular filtration barrier wherein podocytes are considered critical for regulating the passage of plasma proteins into urine.

### Interleukin-6 disrupted the podocyte actin cytoskeleton in a dose-dependent manner

**Figure 7:**
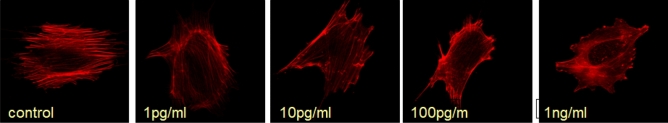
IL-6 causes derangement of the actin cytoskeleton in cultured mouse podocytes. Differentiated podocytes were incubated with IL-6 (0.001–1 ng/mL) for 1 h at 37 °C. Cells were stained for actin filaments using Rhodamine-conjugated phalloidin and observed using confocal microscope. Representative images (×63 objective) show a parallel arrangement of F-actin thick bundles running across the cell in control podocytes. IL-6 (0.001–1 ng/mL) caused change in the thickness, length, and arrangement of actin filaments in a dose-dependent manner. These changes were discernible at 0.001 ng/mL IL-6. A dose-dependent appearance of ‘actin dots’ representing focal adhesions and actin bundling next to the plasma membrane was conspicuous.

A dynamic actin cytoskeleton is critical for maintaining podocyte structure and function in the glomerular filtration barrier. Previously, we showed that injury to podocytes from puromycin, lipopolysaccharide or fluid flow shear stress results in loss of fine F-actin stress fibers and appearance of thick bundles of cortical actin filaments which are surrogate for condensation of the actin cytoskeleton seen in podocytes in vivo^30,31^. Rhodamine-conjugated phalloidin staining of podocytes incubated with IL-6 (0.001–1 ng/mL, 1 h) showed the formation of a cortical ring indicating rearrangement of actin filaments. A more conspicuous finding was a dose-dependent appearance of ‘actin dots’ (Fig. 7). Actin dots represent focal adhesions that suggest actin bundling next to the plasma membrane. The cytoskeletal derangement seen in vitro serves as a useful surrogate for cellular changes that would affect the filtration barrier function.

### Podocytes express both sub-units of IL-6 receptor heterodimer and IL-6 activates the Janus kinase-signal transducer and activator of transcription (JAK-STAT) pathway

**Figure 8:**
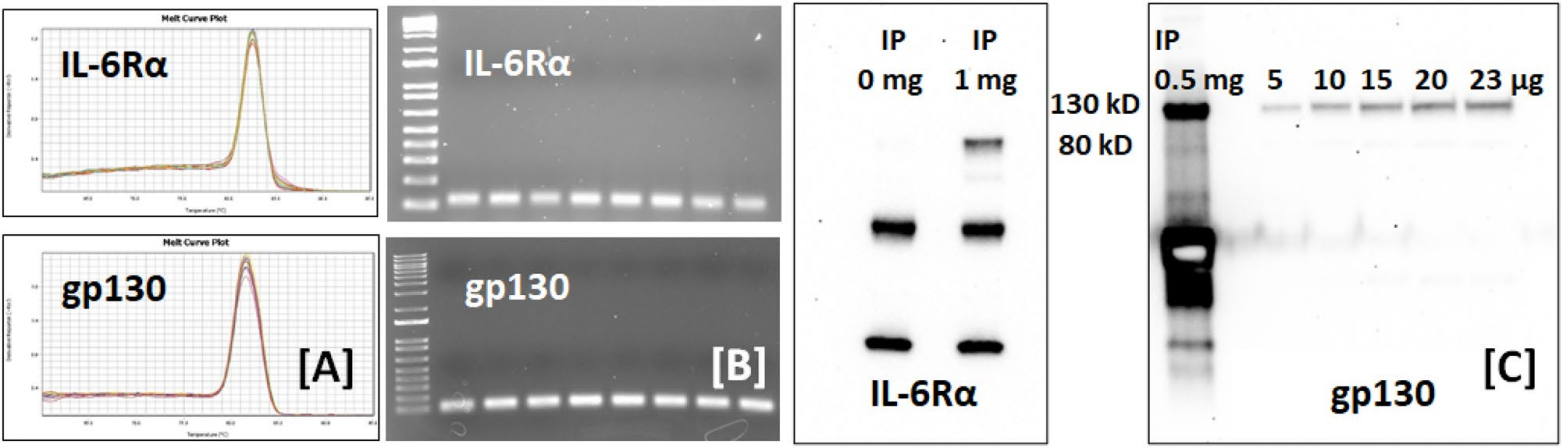
Cultured podocytes express IL-6 receptor sub-units IL-6Rα and gp130. Monolayers of immortalized murine podocytes were used to prepare cell lysate for total RNA and protein expression of IL-6Rα and gp130 as described. (**A**) Melting curve plot using RT-qPCR showed single peaks for IL-6Rα and gp130 each. (**B**) RT-PCR analysis showed single band on 1% agarose gels for IL-6Rα and gp130 each (n = 4 each sample analyzed in duplicates). Western blotting for IL6Rα and gp130 was performed following immunoprecipitation using 1 mg total protein IL-6Rα and 0.5 mg for gp130 (no protein for control). (**C**) Western blot image shows a band at 80 kD demonstrating expression of IL-6Rα and at 130 kD for gp130 in podocytes. Total protein loaded 5, 10, 15, 20 and 23 µg of protein lysate for gp130 expression in the gel. These studies demonstrate both gene and protein expression of IL-6Rα and gp130 in podocytes.

Podocytes are an integral part of the glomerular filtration barrier and play pivotal role in maintaining the barrier to passage of plasma proteins into glomerular filtrate. The IL-6 receptor is a heterodimer consisting of two transmembrane glycoproteins, namely the ligand binding IL-6Rα (CD126) and gp130 (Il6st/gp130/IL6-β/CD130), the signaling subunit shared by several other cytokines of the IL-6 family. Expression of IL-6Rα and gp130 is limited to certain cell types. To document if podocytes express both sub-units of IL-6 receptor heterodimer, we performed gene expression studies in podocytes (n = 4) in duplicate using RT-qPCR with and without melting curve plots. The CT values (mean ± SEM) for IL-6Rα, gp130 and β-actin were 30.09 ± 0.09, 22.06 ± 0.11 and 17.87 ± 0.19, respectively. RT-qPCR results showed a single peak on melting curve plot and subsequent RT-PCR showed a single band on the agarose gel (Fig. 8A,B). The IL-6Rα and gp130 proteins were detectable by Western blotting following immunoprecipitation (Fig. 8C).

**Figure 9:**
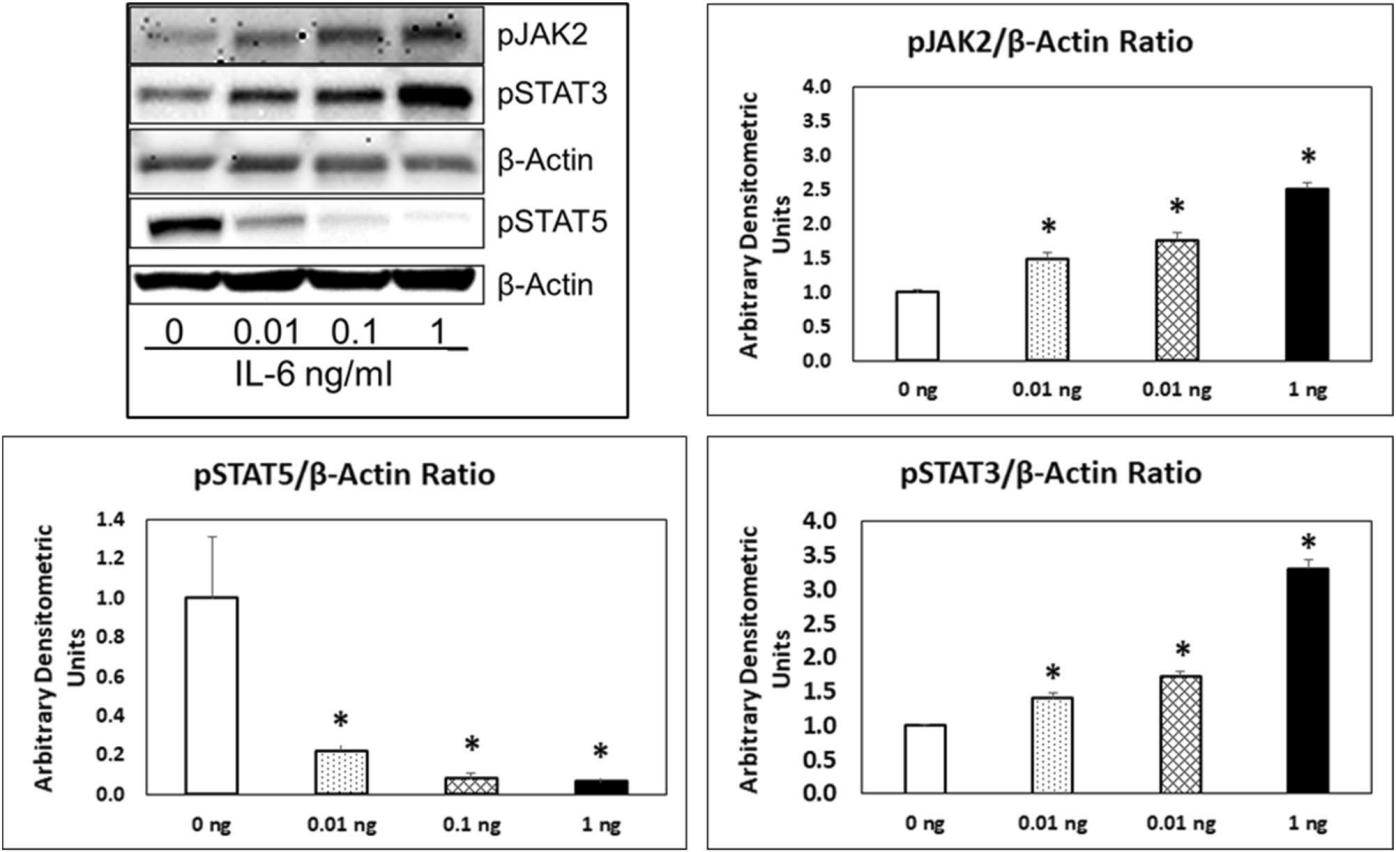
Treatment of podocytes with IL-6 results in upregulation of JAK-STAT pathway. Representative Western blot (n = 4) showing changes in phosphorylation of JAK2, STAT3 and STAT5 in mouse podocytes treated with IL-6 (0.01–1 ng/mL) for 15 min. β-Actin was used as the loading control. The bar graphs show the changes in pJAK2, pSTAT3 and pSTAT5 across the four replicates (*p < 0.05).

Present results demonstrate that podocytes express IL-6 receptor heterodimer and respond to IL-6. Figure 9 shows Western Blot analysis for pJAK2, pSTAT3 and pSTAT5 following treatment of podocytes with 0.01, 0.1 and 1 ng/mL of IL-6 for 15 min. Increased phosphorylation of JAK2 (Tyr1007) and STAT3 (Tyr705) in podocytes was observed. However, phosphorylation of STAT5 (Tyr694) was decreased following exposure to IL-6 highlighting different functions of STAT isoforms.

Taken together, these in vitro results indicate that podocytes respond to IL-6 via its constitutively expressed receptor resulting in JAK-STAT activation, cytoskeletal derangements, and subsequent impairment of the glomerular filtration barrier which is an early event in the onset of proteinuria seen with glomerular dysfunction in CKD.

## Discussion

Recent studies reporting low kidney volume/birth weight ratio, increased incidence of CKD, and increased risk for congenital anomalies in children of obese mothers underscore the significance of intrauterine environment and motivated us to develop this model to study susceptibility to CKD in adult progeny^5–11^. The results summarized above show that prenatal treatment of pregnant mice with IL-6 resulted in newborns with smaller kidneys, lower body weight, kidney morphology indicating accelerated development, upregulated JAK-STAT pathway, altered expression of cell-cycle and apoptosis genes, and increased genomic DNA methylation. Additionally, IL-6 increased albumin permeability of glomeruli ex vivo; deranged the actin cytoskeleton and upregulated JAK-STAT pathway signaling in podocytes in vitro and attenuated the growth of metanephric kidney in vitro. We surmise that elevated levels of IL-6 in chronic inflammation and/or exposure to increased maternal IL-6 during gestation may contribute to greater susceptibility to CKD in the adult offspring.

IL-6 is a pleiotropic cytokine with diverse effects, and its elevated levels are routinely mentioned to signify inflammation associated with pathophysiology of several conditions including CKD and obesity. However, the effect of elevated maternal IL-6 on intrauterine development of the newborn kidney is unclear. Cytokines of the IL-6 family are known to regulate a large number of cellular processes including development. Members of this family include IL-6, Interleukin-11, Interleukin-27, leukemia inhibitory factor, oncostatin M, ciliary neurotrophic factor, cardiotrophin-1, novel neurotrophin-1/B cell stimulating factor-3 or cardiotrophin-like cytokine factor 1 (CLCF1), and neuropoietin. IL-6 is the most extensively studied member of this family of cytokines that primarily activate the JAK-STAT pathway through a receptor complex composed of the transmembrane glycoprotein gp130 (signaling sub-unit) and a ligand-specific protein (ligand binding sub-unit). A third sub-unit protein may also be present for engaging certain cytokines (e.g., CLCF1)^32^. A description of the nature and range of diverse and sometimes opposite effects of IL-6 in different cell types and conditions is beyond the scope of this article but it suffices to say that it engages *cis* and *trans* signaling mechanisms for mediating a large number of biological processes. Here we focused on the influence of elevated maternal IL-6 on the intrauterine development of the kidney.

Our plans for in vivo experiments using pregnant mice to study the effect of IL-6 on intrauterine development of kidneys were encouraged by previously reported observations in animals. These reports showed that among various pro-inflammatory cytokines (IL-1β, TNFα, IL-6), only IL-6 crossed the placental barrier during mid-gestation^16–18^. Additionally, mid-gestation injection of IL-6 resulted in progeny with hypertension during adulthood^16^. Such molecular specificity of the placental barrier and long-term effect of IL-6 prompted us to consider IL-6 as a unique surrogate to study effects of maternal systemic inflammation on kidney development. Serum IL-6 levels reported in pregnant obese women are 1.5- to 2-fold higher compared to non-obese pregnant women^19–21^. In our animal model we wanted to achieve serum levels of IL-6 similar to obese pregnant women. Circulating levels of IL-6/mL are in the low picogram range. We also considered the rapid clearance of injected IL-6 from circulation. Previous studies used 5 or 9 ng/g IL-6 for injection which were orders of magnitude higher than circulating IL-6 in mice^16,18,33^. We calculated that ~ 10 pg/g body weight would be sufficient to transiently increase serum levels for IL-6 without prolonged increase in circulating levels. To keep IL-6 dosing to minimum, it was injected at 48 h intervals. Serum IL-6 levels following intraperitoneal injection in mice at 10 pg/g body weight achieved peak circulating levels at 1 h (~ 60 pg/mL) returning to baseline (~ 20 pg/mL) by 3 h (Multi-plex Array, Bioplex-200, BioRad, Hercules, CA). Thus we refined and advanced the earlier approach by using lower doses of IL-6 (IP,10 pg/g BWT or 1/500–1/900 of previously used amounts) every other day to timed-pregnant mice starting on E12.5^16,18,33^.

Figure 1 summarizes that prenatal treatment of dams with IL-6 resulted in newborns with lower weight, smaller kidneys, and lower kidney weight/body weight ratio. These results are similar to findings described in humans^5^. Body and kidney weights of pups exposed to IL-6 were lower by ~ 20% which is in accord with the in vitro attenuation in the size of metanephroi treated with IL-6 (Fig. 1E). The tissue morphometry (Figs. 3, 4), gene expression (Fig. 5) and DNA methylation in newborn kidney suggest potentially lasting effects of higher levels of maternal IL-6. A consistent, reproducible, and convenient model is needed to investigate the specific role of maternal inflammatory molecules. Current animal models using a high-fat Western diet are associated with marked variability in weight, involve effects of small metabolites outside of inflammation, and tend to breed poorly. IL-6, a pleiotropic cytokine, is highly suitable to model maternal systemic inflammation. This experimental approach enables us to use very small amounts of IL-6 and successful completion of pregnancy in all mice yielding sufficient number of animals for further study and allows for clear interpretation of results for establishing causal relationship. In our animal model we have considered transplacental passage of IL-6, but we need to also consider the independent role of placenta in our animal model.

Our previous work also suggests that intrauterine environmental stress influences the pace of development and organ maturation for postnatal survival. For example, we detected significantly higher serum cortisol level (stress marker) in human preterm neonates who did not develop respiratory distress^34^. Dexamethasone, a synthetic glucocorticoid known to act like a stress hormone, is given to pregnant women at risk for premature delivery to accelerate lung maturity for the preterm child’s survival. In animal models, dexamethasone not only accelerates lung maturation but also results in decreased nephron number in rats, spiny mouse, and sheep^35–38^, and poor response to salt loading or glucose tolerance test on long-term follow up^39,40^. Thus, while intrauterine environmental stress surrogate IL-6, accelerates organ development, it also appears to cause lower nephron number as a trade-off as observed in present experiments. We did not measure serum cortisol in the pregnant dams treated with saline or IL-6 in our current study.

Renal size and birth weight are often used as indicators of nephron endowment. Schreuder et al.^41^ reported decrease in nephron number in natural intrauterine growth retardation (IUGR) or experimental IUGR by bilateral uterine artery ligation in their rat model. In line with observations of Schreuder et al.^41^, a 15–20% decrease in kidney size observed in our experiments implies a lower nephron endowment. Similarly, in humans, birth weight and kidney volume are well-recognized determinants of nephron number and overall kidney function in health and disease. In humans, lower birth weight is associated with smaller kidney size and volume^42^. Also, the mean kidney parenchymal thickness is closely associated with kidney volume, and birth weight^43,44^. Low birth weight (or nephron endowment) is a key factor in the progression of CKD, ESRD, hypertension, albuminuria, and cardiovascular disease in both adults and children^45–49^. Meta-analysis of studies on low birthweight individuals reported 81%, 58%, 79% and 21% increased risk for albuminuria, ESRD, CKD and hypertension, respectively^50–52^. Thus, low birthweight increases the susceptibility to CKD. Thus, low birthweight and low kidney weight with accelerated maturation observed in present studies leads us to postulate that newborns from dams treated with IL-6 will be suitable to study susceptibility to CKD later in life.

CKD is associated with glomerular dysfunction resulting in albuminuria/proteinuria^22,23^, therefore, we also tested the direct effect of IL-6 on the glomerular filtration barrier function. Our results provide evidence for a direct effect of IL-6 on glomerular filtration barrier (Fig. 6), upregulation of JAK-STAT signaling in podocytes (Fig. 9) and derangement of actin cytoskeleton in podocytes (Fig. 7). Podocytes are a key constituent of the glomerulus and their ultra-structural integrity is essential for the glomerular filtration barrier function that regulates passage of plasma proteins into urine. Interdigitating foot processes from podocytes cover the basement membrane over the capillary endothelial cells and form slit pore junctions essential for regulating the passage of plasma proteins. An elaborate and dynamic actin cytoskeleton is critical for maintaining the characteristic cell body and foot processes that maintain the function of podocytes. Derangement of the actin cytoskeleton by IL-6 (Fig. 7) is comparable to that caused by other agents. We have previously shown that puromycin, lipopolysaccharide, prostaglandin E_2_ or fluid flow shear stress induce loss of fine F-actin stress fibers and appearance of thick bundles of cortical actin filaments in podocytes^30,31^. Changes in the podocyte cytoskeleton observed in vitro serve as indicators of impaired filtration barrier function assessed using the albumin permeability (P_alb_) assay using intact glomeruli.

The P_alb_ assay evaluates integrity of the glomerular filtration barrier function in vitro. As shown in Fig. 6, IL-6-induced increase of P_alb_ that was blocked by anti-IL-6 antibody suggesting a direct effect of IL-6 on the filtration barrier corroborating its effect on podocytes. Previously, we and others have used the P_alb_ assay to show the direct effects of cytokines (TNFα, TGFβ1, CLCF1), free radicals, eicosanoids, and other agents on the glomerular filtration barrier function. Using rodent models of proteinuric kidney disease we have demonstrated that increased P_alb_ indicates injury to the glomerular filtration barrier preceding the onset of overt proteinuria^26–29^. Thus, the observed effect of IL-6 on podocyte structure indicates its effect on the glomerular filtration barrier.

The in vitro effects of IL-6 on podocytes and P_alb_ strongly also relate to our ongoing work on CLCF1, a member of the IL-6 family of cytokines, that also utilizes gp130 subunit in a tripartite receptor complex and activates the JAK-STAT signaling pathway. We identified CLCF1 in sera from patients with recurrent focal segmental glomerulosclerosis (FSGS)^53^. Treatment with CLCF1 increased P_alb_ of isolated rat glomeruli that was blocked by anti-CLCF1 antibody or by JAK2 inhibitor BMS911543^54,55^. Moreover, CLCF1 caused derangement of the actin cytoskeleton organization, upregulated phosphorylated JAK2 and STAT3 in cultured podocytes^54–56^. A single injection or chronic infusion of CLCF1 for 28 days increased pSTAT3 in the renal cortex and induced albuminuria^55^. Similarly, present studies demonstrated upregulation of pJAK2 and pSTAT3 in podocytes and in kidneys exposed to IL-6 (Figs. 2, 9). These parallel observations using CLCF1, and IL-6 further strengthen the significance of the JAK-STAT pathway in CKD.

Research on JAK-STAT signaling in CKD has intensified in recent years. JAK-STAT pathway is upregulated in early diabetic nephropathy in animal models and in human kidney tissue^57,58^. Glomerular and tubulointerstitial expression of JAK and STAT is increased, and JAK2 inhibitors mitigate diabetic kidney injury^58–60^. In addition to diabetic nephropathy, an activated JAK-STAT pathway has been reported in UUO, in HIVAN, in nephrotic syndrome, in ADPKD and in IgA glomerulonephritis^61–67^. Thus, upregulation of the JAK-STAT pathway is apparent in CKD of different etiologies.

We believe that there are at least two clinical scenarios where susceptibility for CKD needs special attention as the reason(s) for progression to ESRD in these conditions is still unclear: (a) in adults following kidney donation and (b) in children born with solitary functioning kidney. We recently reviewed the accumulating evidence that living kidney donation increases the risk of ESRD in donors despite stringent screening^68^. The risk for ESRD in donors compared to eligible healthy individuals increases 6.5-fold (USA), 11.4-fold (Norway), and 3.5- to 5.5-fold (Canada), with an overall 8.3-fold increase reported in a meta-analysis of 52 studies^69–72^. There is a cumulative increase in the incidence of ESRD over time post-kidney donation with a reported mean interval of 27.1 ± 9.8 years^73–75^. Similarly, nearly 50% children born with SFK progress to ESRD as young adults^76^. Children with SFK manifest renal injury at a median age of ~ 15 years; 16–26% develop hypertension, 19–21% have proteinuria, and 6–10% have eGFR < 60 mL/min/1.73 m^2^^77–79^.

In a paradigm for developmental origins of health and disease (Barker’s hypothesis), maternal systemic inflammation may be a critical factor in the developing kidney and results in altered fetal programming which becomes consequential in those who develop CKD/ESRD following stress or injury later in life. We envision a ‘two-hit model’ to address the susceptibility to CKD in adults. Our proposed mouse model will be valuable for correlating the intrauterine insult (‘first hit’) with a ‘second hit’ such as dietary stress, unilateral nephrectomy, diabetes, and immune injury that results in the development of adult CKD. As noted above, overactivation of the JAK-STAT pathway observed during development in these studies, and its relevance in kidney diseases of different etiologies in adults is highly significant for future translational studies and for developing intervention(s).

In conclusion, present studies provide a mouse model to further investigate the effects of prenatal exposure to IL-6 as a surrogate of maternal systemic inflammation (e.g., maternal obesity) that results in offspring with small kidneys with accelerated maturation, and JAK-STAT activation. We intend to use this model to study susceptibility to CKD in adult offspring. Such models will prove highly valuable for determining the long-term effects of the growing incidence of maternal obesity, childhood obesity and increased prevalence of obesity and CKD across the globe.

## Material and methods

### Prenatal treatment of pregnant mice with IL-6

Animal studies were approved by the Institutional Animal Care and Use Committee (IACUC), Institutional Biosafety Committee/ Subcommittee on Research Safety (IBC/SRS) and the Research and Development (R&D) Committee at the VA Medical Center, Kansas City, MO, USA. The study was carried out in compliance with the ARRIVE guidelines. All methods were performed in accordance with the relevant guidelines and regulations. Timed-pregnant C57BL/6 mice were obtained from Charles River Laboratories (Indianapolis, IN) and maintained at AAALAC-approved facilities with unrestricted access to food and water under light/dark cycles of 12/12 h. Pregnant mice in the experimental group received IL-6 (10 pg/g body weight [BW]) intra-peritoneally (IP) on alternate days from E12.5 to end of gestation while the control pregnant mice received normal saline. The pups were sacrificed within 6 h of birth and studied on postnatal day P0. Following euthanasia, newborn kidneys were removed by dissection and fixed in 10% formalin, embedded in optimal cutting temperature compound (OCT), or used to isolate total RNA and to prepare total protein lysate. Newborn kidney samples were handled independently or pooled together from a single dam depending on the design of the experiment.

### Light microscopy observation of newborn mouse kidney morphology to identify effects of IL-6 on kidney development

Five micrometer thick sections of formalin-fixed kidneys embedded in paraffin blocks were obtained and stained with hematoxylin and eosin. Kidney tissue sections from 1–2 representative animals from saline (n = 4) or IL-6 treated (n = 4) dams were used for assessing morphology, nephrogenic zone width, mature glomeruli, and pre-tubular aggregates. The nephrogenic zone was defined as the area in the outer renal cortex exhibiting developing nephrons in the form of comma and S-shaped bodies. Kidney sections were viewed at 200× magnification and analyzed using Image J software suite (National Institute of Health and the Laboratory for Optical and Computational Instrumentation at the University of Wisconsin, Madison WI) for measurement of the nephrogenic zone width. Average width for the nephrogenic zone was calculated from five separate randomly measured regions^80,81^. The developing kidney shows glomeruli at different stages of development from comma, S-shape, immature, and mature glomeruli. In developing kidney cap mesenchymal cells aggregate and condense to form a ball of cells near the tip of ureteric bud to form pre-tubular aggregates that may eventually form a mature tubule. A 400 µm × 400 µm grid was used over five separate areas to count the number of mature glomeruli and pre-tubular aggregates per grid.

### Double immunofluorescence staining for podocyte proteins podocin and podocalyxin in newborn mouse kidney glomeruli as indicators of maturation

Four micrometer thick sections of OCT embedded kidneys were double immune stained for podocin and podocalyxin to identify mature glomeruli as described previously^24^. We used rabbit anti-podocin antibody (Catalog # ab50339, Abcam, Cambridge, MA, USA) at 1:500 and goat anti-podocalyxin antibody (Catalog #AF1556, R&D Systems, Minneapolis, MN, USA) at 1:200 with secondary antibodies (at 1:500 dilution) Alexa fluor 488 donkey anti-rabbit (Catalog # A21206) and Alexa Fluor plus 594 Donkey anti-goat (Catalog # A32758) from Invitrogen, Waltham, MA, USA. The number of mature glomeruli expressing both podocalyxin and podocin were counted at 100× from five separate images per animal.

### In vitro organotypic culture to observe the effect of IL-6 on developing metanephroi

Metanephroi were harvested from pregnant dams at post-conception day 13.5 and grown in vitro in medium containing 10 pg/mL IL-6 or without IL-6 (Control) for up to 72 h. Organ cultures were replenished with fresh treatment medium every 24 h. Morphology of metanephric kidneys cultures was photographed at 0, 24, 48 and 72 h using light microscopy at identical magnifications. Metanephroi were outlined manually, and the surface area was calculated by pixel counting. Net growth was determined as the mean percent change in the surface area from before treatment (baseline) to the surface area after treatment. Image J software was used for quantification as described^82^.

### Total RNA isolation, quantitative real time RT-PCR (RT-qPCR) and RT-PCR to evaluate gene expression in podocyte and kidney tissue

Total RNA from podocytes and newborn kidneys was extracted using the Mini Total RNA Purification System from Qiagen (Qiagen, Germantown, MD) Catalog#74104 performed using on-column DNase digestion step. The RNA preparation was then analyzed for quality and quantity by absorbance at 260 and 280 nm using Nanodrop for Nucleic Acid Quantification (Thermo Fisher Scientific, Waltham, MA, USA). The OD260:OD280 absorbance ratio was 1.8–2.0 indicating clean RNA preparations. RNA was aliquoted and stored at − 80 °C. RT-PCR was performed using commercially available primers for IL-6Rα (CD126) Catalog# PPM03027F-200, gp130 (Il6st/gp130/IL6-β/CD130) Catalog# PPM03123C-200 and β-actin (Actb) Catalog# PPM02945B-200 from Qiagen (Qiagen, Germantown, MD, USA). The predicted PCR products were 108, 97 and 154 bp for IL-6Rα, gp130 and β-actin, respectively. The PCR product following RT-PCR was electrophoresed using 1% agarose gel to confirm the size and for single product only.

### PCR Array analysis of cell cycle and apoptosis related genes to determine effect of IL-6 on specific gene expression

Cell cycle and apoptosis pathway genes were evaluated using specific 96-well plate RT^2^ Profiler PCR Arrays from Qiagen (Qiagen, Germantown, MD, USA). Mouse cell cycle (PAMM-020ZA, Qiagen) and mouse apoptosis (PAMM-012Z, Qiagen) panels were used to design these arrays. The RT^2^ Profiler PCR Array included controls for genomic DNA contamination, reverse transcription efficiency, real-time PCR efficiency and housekeeping genes β-glucuronidase (Gusb), glyceraldehyde-3-phosphate dehydrogenase (Gapdh), β-actin (Actb), heat shock protein 90 alpha (cytosolic), class B member (Hsp90ab1) and β-2 microglobulin (B2m). One µg of total RNA was converted to cDNA using Qiagen RT^2^ first strand kit (Qiagen, Catalog# 330404). RT-qPCR analysis of Qiagen RT^2^ Profiler PCR Array was performed using SYBR green with Rox according to the manufacturer’s instructions. All raw data were uploaded to Qiagen data analysis website (https://geneglobe.qiagen.com). Samples from five separate experiments were analyzed.

### Liquid chromatography-mass spectrometry (LC–MS) analysis of total DNA methylation to determine potential epigenetic effects of IL-6

Global DNA methylation studies, a marker of epigenetic changes in fetal DNA, were performed using LC–MS at Zymo Research Corporation (Irvine, CA, USA). Briefly, the newborn kidney tissue was cut into small pieces and sonicated for DNA isolation and analysis. Samples of genomic DNA from each group were hydrolyzed using DNA Degradase Plus Cocktail (Zymo Research). Nucleotides were separated using reverse phase liquid chromatography (UPLC column Eclipse C18, 2.1 × 50 mm, 1.8 um particles, Agilent). The effluent was directed to an ESI source (Agilent Jet Stream) connected to a quadrupole mass spectrometry system (Agilent 6460 QQQ). Each sample was analyzed in triplicates.

### Glomerular albumin permeability (P_alb_) assay in vitro to determine a direct effect of IL-6 on glomerular filtration barrier

Freshly isolated glomeruli from male Sprague Dawley (200–250 g) rats were used to study the changes in glomerular filtration barrier function characteristics using an in vitro assay established in our laboratory. Briefly, kidneys were harvested from rats and renal cortex was cut into approximately 1 mm pieces and pressed through 80-mesh stainless screen followed by passing the filtrate through 120 and 200 mesh screens sequentially. Glomeruli were recovered from atop the 200-mesh screen at room temperature in a physiological buffer solution (pH 7.4) containing 5 gm/dL bovine serum albumin (BSA).

Isolated glomeruli were incubated with the test substance in medium containing 5% BSA for 15 min at 37 °C, transferred to glass coverslips coated with poly-l-lysine (Sigma-Aldrich) and observed using video microscopy. Glomerular images were recorded in the initial incubation medium (5 gm/dL BSA) and 1 min after switching to lower oncotic (1 gm/dL BSA) medium. Oncotic gradient across the capillary wall (5% BSA in the lumen *vs.* 1% in the bathing medium) results in a net fluid influx causing an increase in glomerular volume. Change in glomerular volume (ΔV) due to oncotic gradient calculated from the average of 4 diameters of the video image (ΔV = (V_final_ − V_initial_)/V_initial_ ×100%) and is directly related to the oncotic gradient (ΔΠ) across the capillary wall. Reflection coefficient (σ_alb_) was calculated (σ_alb_ = ΔV_experimental_/ΔV_control_). Convectional permeability to albumin (P_alb_) is defined as (1-σ_alb)_ and describes the movement of albumin consequent to water flow on a unit less scale of 0–1. When σ_alb_ is zero, albumin moves at the same rate as water and P_alb_ is 1.0 indicating damage to the filtration barrier; when σ_alb_ is 1.0, albumin does cross the membrane with water and P_alb_ is zero indicating an intact barrier^26,83^.

### Podocyte cell culture

Conditionally immortalized mouse podocytes containing thermosensitive tsA58 mutant T-antigen (kindly provided by Dr. Peter Mundel) were seeded on collagen-I coated 25 cm^2^ polystyrene flasks and first propagated in RPMI 1640 containing l-glutamine, 10% fetal bovine serum, 100 units/mL penicillin and 0.1 mg/mL streptomycin (Invitrogen, Carlsbad, CA, USA) supplemented with 10 units/mL of γ-interferon (Cell Sciences, Norwood, MA) under permissive conditions at 33 °C with 95% humidity and 5% CO_2_^84^. Cells were then transferred to non-permissive conditions (37 °C without γ-interferon) to induce differentiation. Differentiated podocytes on day 14 were used for experiments outlined below.

### Confocal microscopy to determine IL-6-induced changes in the actin cytoskeleton in podocytes

Podocytes grown on Collagen-I coated coverslips were incubated with 0.001–1 ng/mL IL-6 for 1 h. Cells were washed with PBS (× 2), fixed in 4% paraformaldehyde for 10 min and then incubated with phalloidin conjugated Alexa Fluor 568 dye (1:200 dilution, Invitrogen, Carlsbad, CA) for 30 min in the dark. Cover slips were mounted in 5% *n*-propyl gallate in buffered glycerol (glycerol:PBS, 9:1). Cells were viewed using a Leica confocal microscope (Leica DMI 4000 B) at 561 nm excitation. Laser intensity, gain settings, scaling, individual section depth, and pinhole settings were constant throughout.

### Western blotting to determine protein expression in podocytes and kidney tissue

Cultured podocytes and newborn kidneys were lysed in RIPA buffer containing protease and phosphatase inhibitors. Total protein was quantitated using a bicinchoninic acid protein assay kit (BCA1, Sigma-Aldrich, St. Louis, MO). Immunoprecipitation was carried out by mixing 2 µg antibodies IL-6Rα (SC-374259, Santa Cruz Biotechnology, Dallas, TX) or gp130 (SC-376280, Santa Cruz Biotechnology, Dallas, TX) with 75 µL/mL of Protein G Sepharose beads (2 mg/mL, P3296, Sigma-Aldrich, St. Louis, MO, USA) for 1 h. Centrifuged beads were washed and incubated with 1 mg aliquot of protein lysate for IL-6Rα, 0.5 mg for gp130 and 0 mg (negative control) overnight at 4 °C. Protein bound agarose beads were washed with PBS and resuspended in sample buffer and 20 µL and used for Western blotting as described previously^85–87^. Briefly, proteins were denatured in the sample buffer containing β-mercaptoethanol at 94 °C for 5 min. For other Western blots, total protein (10–20 µg/lane) was electrophoresed in 10% Tris–Glycine gel using SDS-PAGE and transferred to a PVDF membrane, washed with TBST (0.1% Tween-20), and blocked using 5% non-fat milk powder. Antibodies to the following proteins were obtained from Cell Signaling Technology (CST), Danvers, MA, and used at 1:1000 dilution: p-JAK2 (CST catalog# 4406S), p-STAT3 (CST# 9145S) and p-STAT5 (CST# 9359S). β-Actin (Catalog #A5441, Sigma, St. Louis, MO) was used as the loading control. TBST-washed membrane was incubated with HRP-conjugated secondary antibody to each primary antibody. Chemiluminescence reagent (Pierce, Rockford, IL) was added and chemiluminescence was detected using iBright FL100 imaging system (Invitrogen, Waltham, MA, USA). Images were analyzed using Image J program. Samples from 4 replicates for each experimental treatment were analyzed.

### Statistics

We used GraphPad and SPSS 23 statistical software for two group comparisons and graphs. In most of the experiments we had pooled newborn kidneys from a litter as n = 1, but as the effect of the offspring of one litter is statistical dependent among each other, all offspring of one litter were counted as n = 1 and analyzed again for certain analyses. Unpaired t-test on GraphPad was used for two group comparisons. A p value < 0.05 was considered significant. For Qiagen RT2 Profiler PCR Array data analysis, raw data were uploaded to the Qiagen data analysis website (https://geneglobe.qiagen.com), which selects one or more of its five housekeeping genes in the RT2 Profiler PCR Array. It then calculates fold-change as the normalized gene expression 2^(−∆∆CT)^ values in the test sample divided by the normalized gene expression 2^(−∆∆CT)^ values in the control sample. A fold-change values greater than one indicates a positive- or an up-regulation, and values less than one indicate a negative or down-regulation. The p values are calculated based on a Student’s t test of the replicate 2^(−∆∆CT)^ values for each gene in the control group and treatment groups. The p value calculation is based on parametric, unpaired, two-sample equal variance and two-tailed distribution.

## Supplementary Information

Supplementary Information 1.
