# Supplementary material for: A mouse model of prenatal exposure to Interleukin-6 to study the developmental origin of health and disease
Source: Sci Rep. 2021 Jun 24;11:13260. doi: 10.1038/s41598-021-92751-6 (PMC8225793; doi:10.1038/s41598-021-92751-6)

**Supplementary Tables:**

**Supplementary Table 1:** The number of pups and their sex born to six pregnant dams who were treated with saline (Control dams) and those treated with IL-6 (10 pg/g BWT, ip) every other day (E12.5- E20) and used in the analysis.

| **Pups (N)** | **Mother** | **#1** | **#2** | **#3** | **#4** | **#5** | **#6** | **Total** |
| --- | --- | --- | --- | --- | --- | --- | --- | --- |
| All (n) | IL-6 | 4 | 10 | 7 | 11 | 10 | 6 | 48 |
|  | Control | 10 | 7 | 10 | 6 | 10 | 10 | 53 |
|  |  |  |  |  |  |  |  |  |
| Male (n) | IL-6 | 3 | 6 | 3 | 5 | 4 | 3 | 24 |
|  | Control | 4 | 3 | 4 | 4 | 5 | 6 | 26 |
|  |  |  |  |  |  |  |  |  |
| Female (n) | IL-6 | 1 | 4 | 4 | 6 | 6 | 3 | 24 |
|  | Control | 6 | 4 | 6 | 2 | 5 | 4 | 27 |

**Supplementary Table 2: Changes in genes with regulatory effect on cell cycle.** Qiagen’s RT² Profiler PCR Array Gene Expression Analysis (Mouse Cell Cycle Panel PAMM-020ZA) was performed to determine the effect of treating dams with IL-6 on in utero development of kidneys. The genes with altered expression at fold change (FC)>1.5 and p-value <0.05 are shown in red, FC>1.4 and p-value <0.05 are shown in green and genes with FC<1.4 and p-value <0.05 are shown in blue. The roles of these genes in cell cycle are also presented.

| Gene | Description | FC | p-value | G1 Phase and G1/S Transition | S phase and DNA Replication | G2 Phase and G2/M Transition | M Phase | Cell CycleCheckpoint and Cell Cycle Arrest | Regulation of the Cell Cycle | Negative Regulation of Cell Cycle |
| --- | --- | --- | --- | --- | --- | --- | --- | --- | --- | --- |
| Skp2 | S-phase kinase-associated protein 2 (p45) | 1.59 | 0.001636 | G1 Phase and G1/S Transition |  |  |  |  | Regulation of the Cell Cycle |  |
| Cdkn3 | Cyclin-dependent kinase inhibitor 3 | 1.29 | 0.148562 | G1 Phase and G1/S Transition |  | G2 Phase and G2/M Transition |  | Cell CycleCheckpoint and Cell Cycle Arrest |  |  |
| Gpr132 | G protein-coupled receptor 132 | 1.25 | 0.471024 | G1 Phase and G1/S Transition |  |  |  |  |  |  |
| Itgb1 | Integrin beta 1 (fibronectin receptor beta) | 1.11 | 0.304432 | G1 Phase and G1/S Transition |  |  |  |  | Regulation of the Cell Cycle |  |
| Myb | Myeloblastosis oncogene | 1.11 | 0.630180 | G1 Phase and G1/S Transition |  |  |  |  |  |  |
| Slfn1 | Schlafen 1 | 1.03 | 0.709398 | G1 Phase and G1/S Transition |  |  |  | Cell CycleCheckpoint and Cell Cycle Arrest |  |  |
| Cdk6 | Cyclin-dependent kinase 6 | -1.01 | 0.952153 | G1 Phase and G1/S Transition |  |  |  |  | Regulation of the Cell Cycle |  |
| Cdc6 | Cell division cycle 6 homolog | 1.51 | 0.001708 |  | S phase and DNA Replication |  | M Phase |  | Regulation of the Cell Cycle |  |
| Cdc7 | Cell division cycle 7 | 1.78 | 0.002318 |  | S phase and DNA Replication |  |  |  |  |  |
| Mcm2 | Minichromosome maintenance deficient 2 mitotin | 1.34 | 0.017567 |  | S phase and DNA Replication |  |  |  |  |  |
| Mki67 | Antigen identified by monoclonal antibody Ki 67 | 1.21 | 0.016439 |  | S phase and DNA Replication |  |  |  |  |  |
| Mcm4 | Minichromosome maintenance deficient 4 homolog | 1.28 | 0.183218 |  | S phase and DNA Replication |  |  |  |  |  |
| Rad17 | RAD17 homolog | 1.27 | 0.162089 |  | S phase and DNA Replication |  |  |  |  |  |
| Rad51 | RAD51 homolog | 1.26 | 0.435150 |  | S phase and DNA Replication |  |  |  |  |  |
| Msh2 | MutS homolog 2 | 1.18 | 0.469624 |  | S phase and DNA Replication |  |  | Cell CycleCheckpoint and Cell Cycle Arrest |  |  |
| Mre11a | Meiotic recombination 11 homolog A | 1.16 | 0.282174 |  | S phase and DNA Replication |  |  |  |  |  |
| Mcm3 | Minichromosome maintenance deficient 3 | 1.12 | 0.305391 |  | S phase and DNA Replication |  |  |  |  |  |
| Chek1 | Checkpoint kinase 1 homolog | 1.77 | 0.006770 |  |  | G2 Phase and G2/M Transition |  | Cell CycleCheckpoint and Cell Cycle Arrest |  |  |
| Birc5 | Baculoviral IAP repeat-containing 5 | 1.14 | 0.217886 |  |  | G2 Phase and G2/M Transition |  |  |  |  |
| Ppm1d | Protein phosphatase 1D magnesium-dependent, delta isoform | -1.11 | 0.333025 |  |  | G2 Phase and G2/M Transition |  | Cell CycleCheckpoint and Cell Cycle Arrest |  |  |
| Ccna1 | Cyclin A1 | 1.93 | 0.000033 |  |  |  | M Phase |  | Regulation of the Cell Cycle |  |
| Cdk1 | Cyclin-dependent kinase 1 | 1.19 | 0.012966 |  |  |  | M Phase | Cell CycleCheckpoint and Cell Cycle Arrest | Regulation of the Cell Cycle |  |
| Cdk2 | Cyclin-dependent kinase 2 | 1.27 | 0.056964 |  |  |  | M Phase |  |  |  |
| Cdc25a | Cell division cycle 25 homolog A | 1.25 | 0.052632 |  |  |  | M Phase |  |  |  |
| Ccnb1 | Cyclin B1 | 1.22 | 0.069380 |  |  |  | M Phase |  | Regulation of the Cell Cycle |  |
| Cdc25c | Cell division cycle 25 homolog C | 1.21 | 0.145338 |  |  |  | M Phase | Cell CycleCheckpoint and Cell Cycle Arrest | Regulation of the Cell Cycle |  |
| Cdc20 | Cell division cycle 20 homolog | 1.19 | 0.242925 |  |  |  | M Phase |  | Regulation of the Cell Cycle |  |
| Nek2 | NIMA (never in mitosis gene a)-related expressed kinase 2 | 1.17 | 0.101014 |  |  |  | M Phase |  |  |  |
| Stag1 | Stromal antigen 1 | 1.17 | 0.262862 |  |  |  | M Phase |  |  |  |
| Brca2 | Breast cancer 2 | 1.16 | 0.366720 |  |  |  | M Phase | Cell CycleCheckpoint and Cell Cycle Arrest | Regulation of the Cell Cycle |  |
| Terf1 | Telomeric repeat binding factor 1 | 1.16 | 0.165239 |  |  |  | M Phase |  |  |  |
| Shc1 | Src homology 2 domain-containing transforming protein C1 | 1.11 | 0.179902 |  |  |  | M Phase |  | Regulation of the Cell Cycle |  |
| Wee1 | WEE 1 homolog 1 | 1.09 | 0.397071 |  |  |  | M Phase |  |  |  |
| Ran | RAN, member RAS oncogene family | 1.05 | 0.902659 |  |  |  | M Phase |  | Regulation of the Cell Cycle |  |
| Smc1a | Structural maintenance of chromosomes 1A | 1.02 | 0.846389 |  |  |  | M Phase | Cell CycleCheckpoint and Cell Cycle Arrest |  |  |
| Rad21 | RAD21 homolog | 1.00 | 0.997181 |  |  |  | M Phase |  |  |  |
| Stmn1 | Stathmin 1 | 1.00 | 0.986544 |  |  |  | M Phase |  |  |  |
| Aurkb | Aurora kinase B | -1.07 | 0.415070 |  |  |  | M Phase |  |  |  |
| Cdk5rap1 | CDK5 regulatory subunit associated protein 1 | 1.59 | 0.031080 |  |  |  |  | Cell CycleCheckpoint and Cell Cycle Arrest |  |  |
| Chek2 | CHK2 checkpoint homolog | 1.54 | 0.004929 |  |  |  |  | Cell CycleCheckpoint and Cell Cycle Arrest |  |  |
| Atr | Ataxia telangiectasia and rad3 related | 1.46 | 0.000461 |  |  |  |  | Cell CycleCheckpoint and Cell Cycle Arrest | Regulation of the Cell Cycle |  |
| Notch2 | Notch gene homolog 2 | 1.36 | 0.024446 |  |  |  |  | Cell CycleCheckpoint and Cell Cycle Arrest |  |  |
| Gadd45a | Growth arrest and DNA-damage-inducible 45 alpha | -1.25 | 0.028246 |  |  |  |  | Cell CycleCheckpoint and Cell Cycle Arrest | Regulation of the Cell Cycle |  |
| Cks1b | CDC28 protein kinase 1b | 1.34 | 0.055694 |  |  |  |  | Cell CycleCheckpoint and Cell Cycle Arrest | Regulation of the Cell Cycle |  |
| Cdkn2a | Cyclin-dependent kinase inhibitor 2A | 1.31 | 0.323688 |  |  |  |  | Cell CycleCheckpoint and Cell Cycle Arrest |  | Negative Regulation of Cell Cycle |
| Pkd1 | Polycystic kidney disease 1 homolog | 1.23 | 0.079026 |  |  |  |  | Cell CycleCheckpoint and Cell Cycle Arrest |  |  |
| Tsg101 | Tumor susceptibility gene 101 | 1.22 | 0.314301 |  |  |  |  | Cell CycleCheckpoint and Cell Cycle Arrest |  |  |
| Casp3 | Caspase 3 | 1.16 | 0.227874 |  |  |  |  | Cell CycleCheckpoint and Cell Cycle Arrest |  | Negative Regulation of Cell Cycle |
| Nbn | Nibrin | 1.16 | 0.309443 |  |  |  |  | Cell CycleCheckpoint and Cell Cycle Arrest |  |  |
| Mad2l1 | MAD2 mitotic arrest deficient-like 1 | 1.15 | 0.466215 |  |  |  |  | Cell CycleCheckpoint and Cell Cycle Arrest |  |  |
| Hus1 | Hus1 homolog | 1.13 | 0.634831 |  |  |  |  | Cell CycleCheckpoint and Cell Cycle Arrest |  |  |
| Ddit3 | DNA-damage inducible transcript 3 | 1.11 | 0.292338 |  |  |  |  | Cell CycleCheckpoint and Cell Cycle Arrest |  |  |
| Rad9a | RAD9 homolog | 1.09 | 0.380791 |  |  |  |  | Cell CycleCheckpoint and Cell Cycle Arrest | Regulation of the Cell Cycle |  |
| Sfn | Stratifin | 1.09 | 0.477049 |  |  |  |  | Cell CycleCheckpoint and Cell Cycle Arrest | Regulation of the Cell Cycle |  |
| Pmp22 | Peripheral myelin protein 22 | 1.05 | 0.435895 |  |  |  |  | Cell CycleCheckpoint and Cell Cycle Arrest |  |  |
| Rb1 | Retinoblastoma 1 | 1.01 | 0.962473 |  |  |  |  | Cell CycleCheckpoint and Cell Cycle Arrest | Regulation of the Cell Cycle |  |
| Cdkn1b | Cyclin-dependent kinase inhibitor 1B | 1.01 | 0.892612 |  |  |  |  | Cell CycleCheckpoint and Cell Cycle Arrest |  |  |
| Cdkn2b | Cyclin-dependent kinase inhibitor 2B (p15, inhibits CDK4) | -1.01 | 0.957961 |  |  |  |  | Cell CycleCheckpoint and Cell Cycle Arrest |  | Negative Regulation of Cell Cycle |
| Mdm2 | Transformed mouse 3T3 cell double minute 2 | -1.02 | 0.907647 |  |  |  |  | Cell CycleCheckpoint and Cell Cycle Arrest |  |  |
| Cdkn1a | Cyclin-dependent kinase inhibitor 1A (P21) | -1.03 | 0.630500 |  |  |  |  | Cell CycleCheckpoint and Cell Cycle Arrest | Regulation of the Cell Cycle |  |
| Dst | Dystonin | -1.03 | 0.909045 |  |  |  |  | Cell CycleCheckpoint and Cell Cycle Arrest |  |  |
| Ccna2 | Cyclin A2 | 1.46 | 0.033909 |  |  |  |  |  | Regulation of the Cell Cycle |  |
| Ccnd2 | Cyclin D2 | 1.42 | 0.000871 |  |  |  |  |  | Regulation of the Cell Cycle |  |
| Aurka | Aurora kinase A | 1.40 | 0.012372 |  |  |  |  |  | Regulation of the Cell Cycle |  |
| Ccnd3 | Cyclin D3 | 1.17 | 0.017355 |  |  |  |  |  | Regulation of the Cell Cycle |  |
| Ccnf | Cyclin F | 1.27 | 0.123979 |  |  |  |  |  | Regulation of the Cell Cycle |  |
| Ccnd1 | Cyclin D1 | 1.26 | 0.122363 |  |  |  |  |  | Regulation of the Cell Cycle |  |
| Cdk4 | Cyclin-dependent kinase 4 | 1.25 | 0.051467 |  |  |  |  |  | Regulation of the Cell Cycle |  |
| Tfdp1 | Transcription factor Dp 1 | 1.24 | 0.087735 |  |  |  |  |  | Regulation of the Cell Cycle |  |
| Ccnb2 | Cyclin B2 | 1.21 | 0.135610 |  |  |  |  |  | Regulation of the Cell Cycle |  |
| Bcl2 | B-cell leukemia/lymphoma 2 | 1.17 | 0.114706 |  |  |  |  |  | Regulation of the Cell Cycle |  |
| Ccnc | Cyclin C | 1.17 | 0.354645 |  |  |  |  |  | Regulation of the Cell Cycle |  |
| Ccne1 | Cyclin E1 | 1.16 | 0.086679 |  |  |  |  |  | Regulation of the Cell Cycle |  |
| E2f3 | E2F transcription factor 3 | 1.15 | 0.098724 |  |  |  |  |  | Regulation of the Cell Cycle |  |
| E2f1 | E2F transcription factor 1 | 1.12 | 0.441350 |  |  |  |  |  | Regulation of the Cell Cycle |  |
| Abl1 | C-abl oncogene 1, non-receptor tyrosine kinase | 1.09 | 0.339285 |  |  |  |  |  | Regulation of the Cell Cycle |  |
| E2f2 | E2F transcription factor 2 | 1.08 | 0.647215 |  |  |  |  |  | Regulation of the Cell Cycle |  |
| E2f4 | E2F transcription factor 4 | -1.04 | 0.669829 |  |  |  |  |  | Regulation of the Cell Cycle |  |
| Atm | Ataxia telangiectasia mutated homolog | 2.29 | 0.000073 |  |  |  |  |  |  | Negative Regulation of Cell Cycle |
| Trp63 | Transformation related protein 63 | 1.73 | 0.000648 |  |  |  |  |  |  | Negative Regulation of Cell Cycle |
| Brca1 | Breast cancer 1 | 1.17 | 0.099873 |  |  |  |  |  |  | Negative Regulation of Cell Cycle |
| Rbl1 | Retinoblastoma-like 1 (p107) | 1.17 | 0.301790 |  |  |  |  |  |  | Negative Regulation of Cell Cycle |
| Rbl2 | Retinoblastoma-like 2 | 1.15 | 0.204687 |  |  |  |  |  |  | Negative Regulation of Cell Cycle |
| Trp53 | Transformation related protein 53 | -1.00 | 0.919542 |  |  |  |  |  |  | Negative Regulation of Cell Cycle |

**Supplementary Table 3: Changes in genes with regulatory role(s) in apoptosis.** Qiagen’s RT² Profiler PCR Array Gene Expression Analysis (Mouse Apoptosis Panel PAMM-012ZA) was performed to determine the effect of treating dams with IL-6 on in utero development of kidneys. The genes with altered expression at fold change (FC)>1.5 and p-value <0.05 are shown in red, FC>1.4 and p-value <0.05 are shown in green and genes with FC<1.4 and p-value <0.05 are shown in blue. The roles of these genes in apoptosis are also presented.

| Gene | Description | FC | p-value | Induction of Apoptosis | Anti-Apoptotic | Death Domain Proteins/ DNA Damage | Caspases | Positive Regulation | Negative Regulation |
| --- | --- | --- | --- | --- | --- | --- | --- | --- | --- |
| Trp63 | Transformation related protein 63 | 1.63 | 0.001611 | Induction of Apoptosis | Anti-Apoptotic | DNA Damage |  |  |  |
| Bnip3l | BCL2/adenovirus E1B interacting protein 3-like | 1.15 | 0.138398 | Induction of Apoptosis | Anti-Apoptotic |  |  | Positive Regulation | Negative Regulation |
| Bax | Bcl2-associated X protein | 1.10 | 0.267255 | Induction of Apoptosis | Anti-Apoptotic |  | Caspase Activators | Positive Regulation |  |
| Dapk1 | Death associated protein kinase 1 | 1.07 | 0.317595 | Induction of Apoptosis | Anti-Apoptotic | Death Domain Proteins |  |  | Negative Regulation |
| Cflar | CASP8 and FADD-like apoptosis regulator | 1.01 | 0.870972 | Induction of Apoptosis | Anti-Apoptotic |  | Caspases |  | Negative Regulation |
| Bnip3 | BCL2/adenovirus E1B interacting protein 3 | -1.07 | 0.529256 | Induction of Apoptosis | Anti-Apoptotic |  |  | Positive Regulation | Negative Regulation |
| Tnf | Tumor necrosis factor | -1.16 | 0.273765 | Induction of Apoptosis | Anti-Apoptotic |  |  | Positive Regulation |  |
| Fas | Fas (TNF receptor superfamily member 6) | -1.20 | 0.068531 | Induction of Apoptosis | Anti-Apoptotic |  |  |  | Negative Regulation |
| Fasl | Fas ligand (TNF superfamily, member 6) | 1.47 | 0.006512 | Induction of Apoptosis |  |  |  | Positive Regulation |  |
| Pycard | PYD and CARD domain containing | 1.35 | 0.004527 | Induction of Apoptosis |  |  | Caspases/Caspase Activators | Positive Regulation |  |
| Bid | BH3 interacting domain death agonist | 1.32 | 0.026291 | Induction of Apoptosis |  |  |  | Positive Regulation |  |
| Fadd | Fas (TNFRSF6)-associated via death domain | 1.27 | 0.024924 | Induction of Apoptosis |  | Death Domain Proteins |  | Positive Regulation |  |
| Dffa | DNA fragmentation factor, alpha subunit | 1.26 | 0.016719 | Induction of Apoptosis |  |  |  |  | Negative Regulation |
| Mapk1 | Mitogen-activated protein kinase 1 | 1.16 | 0.046700 | Induction of Apoptosis |  |  |  |  |  |
| Diablo | Diablo homolog (Drosophila) | 1.15 | 0.026748 | Induction of Apoptosis |  |  |  |  |  |
| Casp4 | Caspase 4, apoptosis-related cysteine peptidase | -1.27 | 0.006693 | Induction of Apoptosis |  |  | Caspases | Positive Regulation |  |
| Bad | BCL2-associated agonist of cell death | 1.24 | 0.097832 | Induction of Apoptosis |  |  |  | Positive Regulation |  |
| Casp2 | Caspase 2 | 1.23 | 0.084075 | Induction of Apoptosis |  |  | Caspases | Positive Regulation |  |
| Bak1 | BCL2-antagonist/killer 1 | 1.18 | 0.084680 | Induction of Apoptosis |  |  |  | Positive Regulation |  |
| Bok | BCL2-related ovarian killer protein | 1.16 | 0.226865 | Induction of Apoptosis |  |  |  |  |  |
| Casp6 | Caspase 6 | 1.14 | 0.184161 | Induction of Apoptosis |  |  | Caspases | Positive Regulation |  |
| Cidea | Cell death-inducing DNA fragmentation factor, alpha subunit-like effector A | 1.11 | 0.475108 | Induction of Apoptosis |  | DNA Damage |  |  | Negative Regulation |
| Cradd | CASP2 and RIPK1 domain containing adaptor with death domain | 1.08 | 0.350130 | Induction of Apoptosis |  | Death Domain Proteins | Caspases | Positive Regulation |  |
| Casp3 | Caspase 3 | 1.08 | 0.228361 | Induction of Apoptosis |  |  | Caspases |  | Negative Regulation |
| Tnfsf10 | Tumor necrosis factor (ligand) superfamily, member 10 | 1.08 | 0.442496 | Induction of Apoptosis |  |  |  | Positive Regulation |  |
| Bcl10 | B-cell leukemia/lymphoma 10 | 1.07 | 0.477526 | Induction of Apoptosis |  |  |  |  | Negative Regulation |
| Bcl2l11 | BCL2-like 11 (apoptosis facilitator) | 1.06 | 0.459743 | Induction of Apoptosis |  |  |  | Positive Regulation |  |
| Trp53 | Transformation related protein 53 | 1.05 | 0.358972 | Induction of Apoptosis |  | DNA Damage | Caspase Activators | Positive Regulation | Negative Regulation |
| Cideb | Cell death-inducing DNA fragmentation factor, alpha subunit-like effector B | 1.05 | 0.477138 | Induction of Apoptosis |  | DNA Damage |  | Positive Regulation |  |
| Casp1 | Caspase 1 | 1.05 | 0.478008 | Induction of Apoptosis |  |  | Caspases/Caspase Activators | Positive Regulation |  |
| Casp8 | Caspase 8 | 1.04 | 0.609851 | Induction of Apoptosis |  |  | Caspases | Positive Regulation |  |
| Casp12 | Caspase 12 | 1.02 | 0.838238 | Induction of Apoptosis |  |  | Caspases |  |  |
| Tnfrsf10b | Tumor necrosis factor receptor superfamily, member 10b | 1.01 | 0.928521 | Induction of Apoptosis |  | Death Domain Proteins | Caspase Activators | Positive Regulation |  |
| Nod1 | Nucleotide-binding oligomerization domain containing 1 | 1.00 | 0.965289 | Induction of Apoptosis |  |  | Caspase Activators | Positive Regulation |  |
| Traf3 | Tnf receptor-associated factor 3 | -1.01 | 0.928323 | Induction of Apoptosis |  |  |  | Positive Regulation |  |
| Trp53bp2 | Transformation related protein 53 binding protein 2 | -1.01 | 0.759163 | Induction of Apoptosis |  |  |  | Positive Regulation |  |
| Casp14 | Caspase 14 | -1.07 | 0.774730 | Induction of Apoptosis |  |  | Caspases | Positive Regulation |  |
| Dffb | DNA fragmentation factor, beta subunit | -1.09 | 0.379627 | Induction of Apoptosis |  |  |  |  |  |
| Trp73 | Transformation related protein 73 | -1.10 | 0.369910 | Induction of Apoptosis |  | DNA Damage |  |  | Negative Regulation |
| Cd70 | CD70 antigen | -1.18 | 0.121717 | Induction of Apoptosis |  |  |  | Positive Regulation |  |
| Atf5 | Activating transcription factor 5 | 1.54 | 0.000052 |  | Anti-Apoptotic |  |  |  |  |
| Il10 | Interleukin 10 | -1.76 | 0.003926 |  | Anti-Apoptotic |  |  |  |  |
| Xiap | X-linked inhibitor of apoptosis | 1.40 | 0.001350 |  | Anti-Apoptotic |  | Caspase Inhibitors |  | Negative Regulation |
| Bcl2l10 | Bcl2-like 10 | -1.38 | 0.000557 |  | Anti-Apoptotic |  | Caspase Activators |  | Negative Regulation |
| Polb | Polymerase (DNA directed), beta | 1.27 | 0.065123 |  | Anti-Apoptotic |  |  |  |  |
| Lhx4 | LIM homeobox protein 4 | 1.19 | 0.183309 |  | Anti-Apoptotic |  |  |  |  |
| Cd40lg | CD40 ligand | 1.18 | 0.127838 |  | Anti-Apoptotic |  |  |  | Negative Regulation |
| Birc5 | Baculoviral IAP repeat-containing 5 | 1.17 | 0.082499 |  | Anti-Apoptotic |  | Caspase Inhibitors |  |  |
| Bcl2l2 | Bcl2-like 2 | 1.15 | 0.140516 |  | Anti-Apoptotic |  |  |  | Negative Regulation |
| Api5 | Apoptosis inhibitor 5 | 1.15 | 0.069140 |  | Anti-Apoptotic |  |  |  |  |
| Naip2 | NLR family, apoptosis inhibitory protein 2 | 1.15 | 0.323100 |  | Anti-Apoptotic |  |  |  |  |
| Dad1 | Defender against cell death 1 | 1.11 | 0.071596 |  | Anti-Apoptotic |  |  |  |  |
| Akt1 | Thymoma viral proto-oncogene 1 | 1.10 | 0.107520 |  | Anti-Apoptotic |  |  | Positive Regulation |  |
| Bag1 | Bcl2-associated athanogene 1 | 1.10 | 0.234052 |  | Anti-Apoptotic |  |  |  | Negative Regulation |
| Bcl2 | B-cell leukemia/lymphoma 2 | 1.09 | 0.442232 |  | Anti-Apoptotic |  |  |  | Negative Regulation |
| Bnip2 | BCL2/adenovirus E1B interacting protein 2 | 1.08 | 0.407932 |  | Anti-Apoptotic |  |  |  | Negative Regulation |
| Nfkb1 | Nuclear factor of kappa light polypeptide gene enhancer in B-cells 1, p105 | 1.07 | 0.139942 |  | Anti-Apoptotic | Death Domain Proteins |  |  |  |
| Prdx2 | Peroxiredoxin 2 | 1.07 | 0.454758 |  | Anti-Apoptotic |  |  |  |  |
| Nol3 | Nucleolar protein 3 (apoptosis repressor with CARD domain) | 1.03 | 0.767460 |  | Anti-Apoptotic |  |  |  | Negative Regulation |
| Mcl1 | Myeloid cell leukemia sequence 1 | 1.01 | 0.784785 |  | Anti-Apoptotic |  |  |  | Negative Regulation |
| Nme5 | Non-metastatic cells 5, protein expressed in (nucleoside-diphosphate kinase) | -1.05 | 0.380486 |  | Anti-Apoptotic |  |  |  |  |
| Naip1 | NLR family, apoptosis inhibitory protein 1 | -1.06 | 0.859907 |  | Anti-Apoptotic |  |  |  |  |
| Bcl2l1 | Bcl2-like 1 | -1.07 | 0.494834 |  | Anti-Apoptotic |  |  |  | Negative Regulation |
| Birc3 | Baculoviral IAP repeat-containing 3 | -1.07 | 0.473890 |  | Anti-Apoptotic |  |  |  | Negative Regulation |
| Bag3 | Bcl2-associated athanogene 3 | -1.12 | 0.097465 |  | Anti-Apoptotic |  |  |  | Negative Regulation |
| Igf1r | Insulin-like growth factor I receptor | -1.13 | 0.246629 |  | Anti-Apoptotic |  |  |  |  |
| Tnfrsf11b | Tumor necrosis factor receptor superfamily, member 11b (osteoprotegerin) | 2.03 | 0.000013 |  |  | Death Domain Proteins |  |  |  |
| Ripk1 | Receptor (TNFRSF)-interacting serine-threonine kinase 1 | 1.21 | 0.039765 |  |  | Death Domain Proteins |  |  |  |
| Tnfrsf1a | Tumor necrosis factor receptor superfamily, member 1a | -1.14 | 0.167746 |  |  | Death Domain Proteins |  |  |  |
| Apaf1 | Apoptotic peptidase activating factor 1 | 1.20 | 0.104279 |  |  |  | Caspase Activators |  |  |
| Aifm1 | Apoptosis-inducing factor, mitochondrion-associated 1 | 1.13 | 0.225708 |  |  |  | Caspase Activators |  |  |
| Casp7 | Caspase 7 | 1.13 | 0.078787 |  |  |  | Caspases |  |  |
| Casp9 | Caspase 9 | 1.10 | 0.136331 |  |  |  | Caspases/Caspase Activators |  |  |
| Traf1 | Tnf receptor-associated factor 1 | 1.56 | 0.000064 |  |  |  |  | Positive Regulation |  |
| Traf2 | Tnf receptor-associated factor 2 | 1.22 | 0.004307 |  |  |  |  | Positive Regulation |  |
| Gadd45a | Growth arrest and DNA-damage-inducible 45 alpha | -1.22 | 0.027658 |  |  |  |  | Positive Regulation |  |
| Card10 | Caspase recruitment domain family, member 10 | 1.26 | 0.083801 |  |  |  |  | Positive Regulation |  |
| Cd40 | CD40 antigen | 1.13 | 0.323868 |  |  |  |  | Positive Regulation |  |
| Abl1 | C-abl oncogene 1, non-receptor tyrosine kinase | 1.09 | 0.487609 |  |  |  |  | Positive Regulation |  |
| Anxa5 | Annexin A5 | 1.04 | 0.575557 |  |  |  |  | Positive Regulation |  |
| Ltbr | Lymphotoxin B receptor | -1.02 | 0.954270 |  |  |  |  | Positive Regulation |  |
| Tnfsf12 | Tumor necrosis factor (ligand) superfamily, member 12 | -1.07 | 0.607043 |  |  |  |  | Positive Regulation |  |
| Birc2 | Baculoviral IAP repeat-containing 2 | 1.08 | 0.328237 |  |  |  |  |  | Negative Regulation |
| Bcl2a1a | B-cell leukemia/lymphoma 2 related protein A1a | -1.05 | 0.402357 |  |  |  |  |  | Negative Regulation |

**Supplementary Figures:**

**Supplementary Figure S1:** IL-6 treatment alters kidney growth in vivo following treatment with IL-6 (10 pg/g BWT, ip) every other day (E12.5- E20). In the current analyses all the litter from each dam was averaged for body weight and kidney weight as a single datapoint as n=1. Box plots show the distribution of body weights (left panel) and kidneys weight (right panel) by sex from n=6 dams treated with either saline or IL-6.


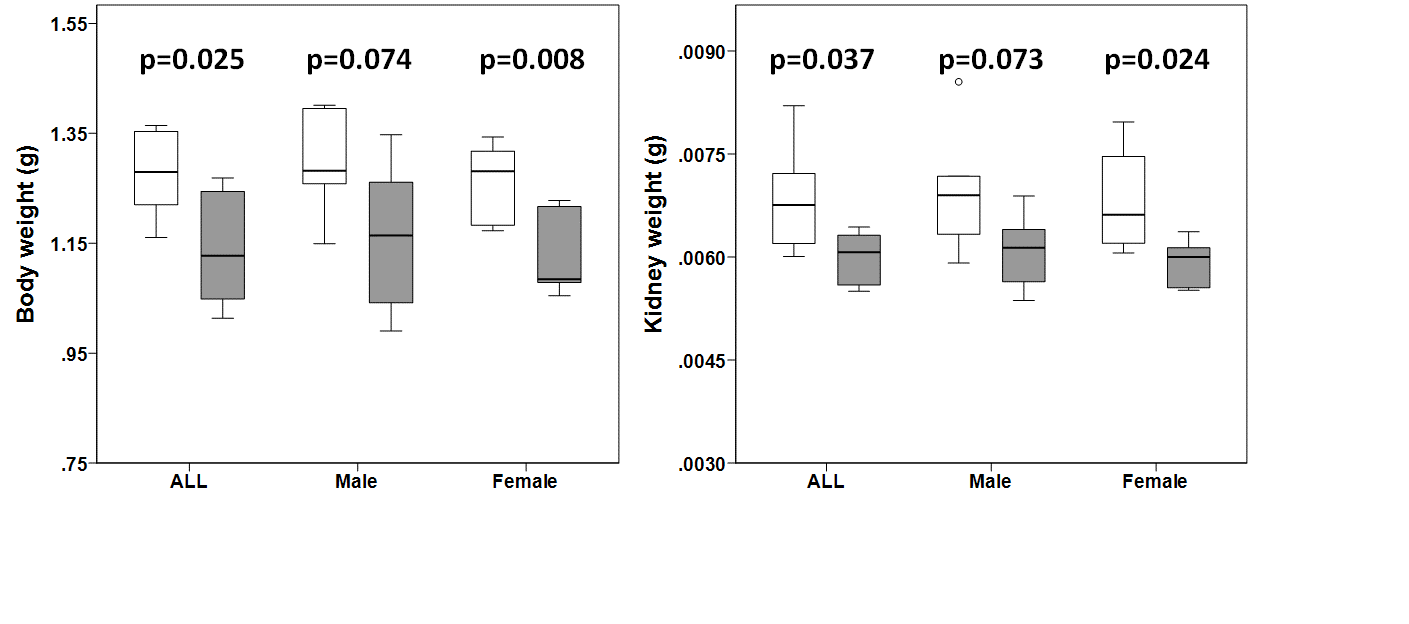


**Supplementary Western blot gels for the submitted Figures 2, 8 and 9.**

**Submitted Figure 2**


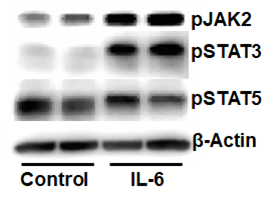


**Supplementary Gel blot for Figure 2**

These blots were cut prior to hybridization. These western blots were done few years ago and these are the only images which we could retrieve from our old computerized system.

**pSTAT3 pJAK2 pSTAT5**


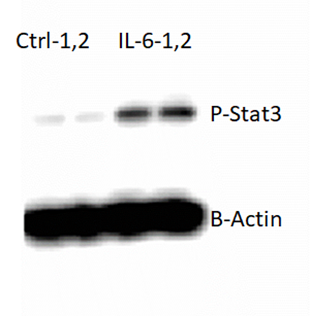

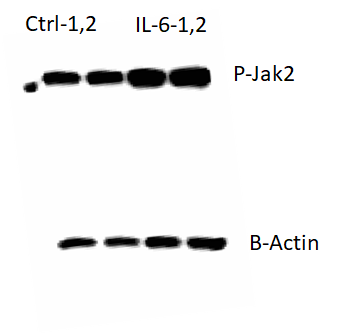

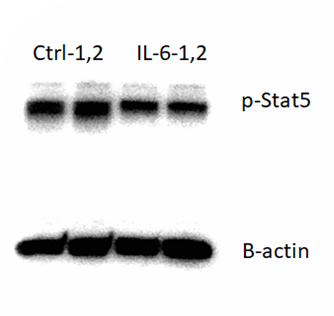


**Submitted Figure 8: The original figure is the whole blot.**


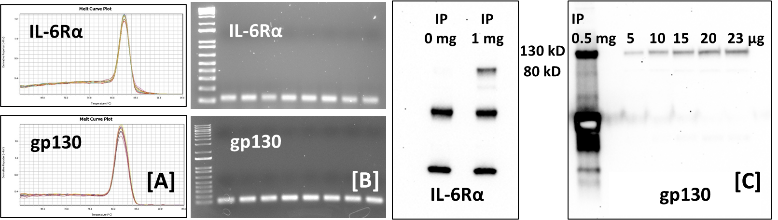


**Supplementary Gel blot for Figure 8**


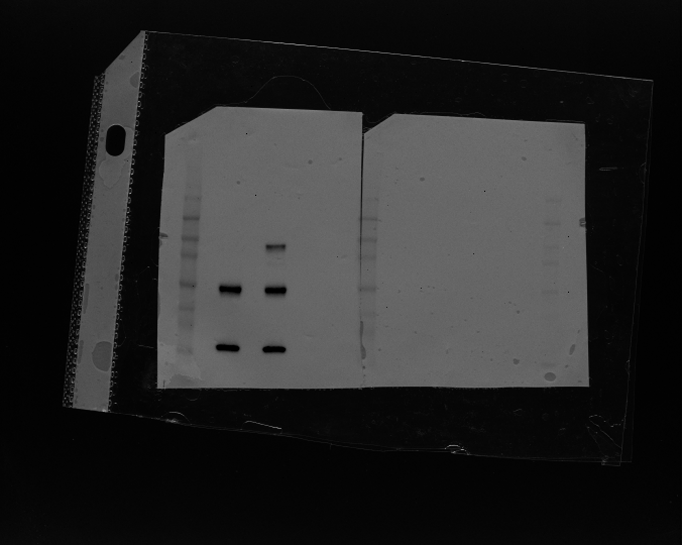

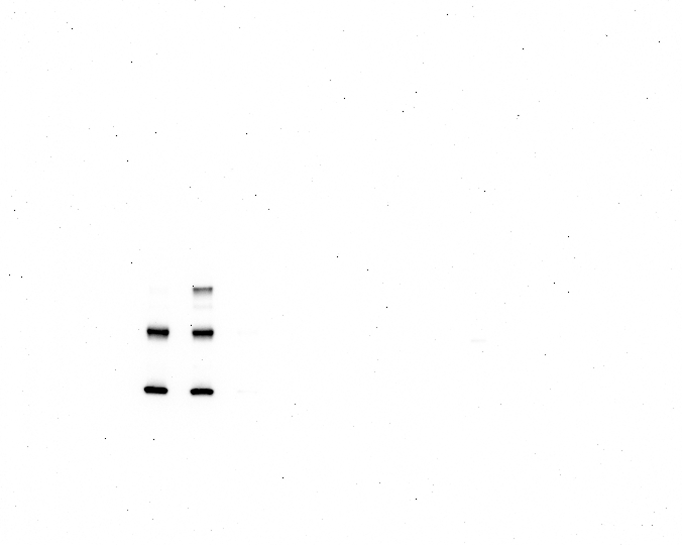

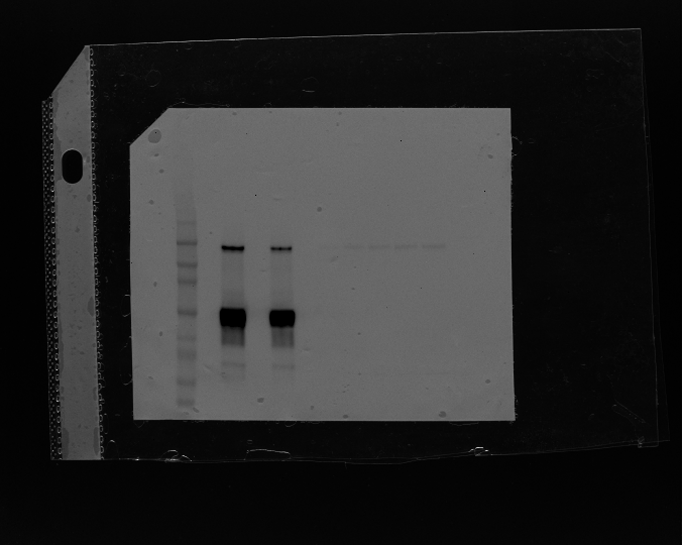

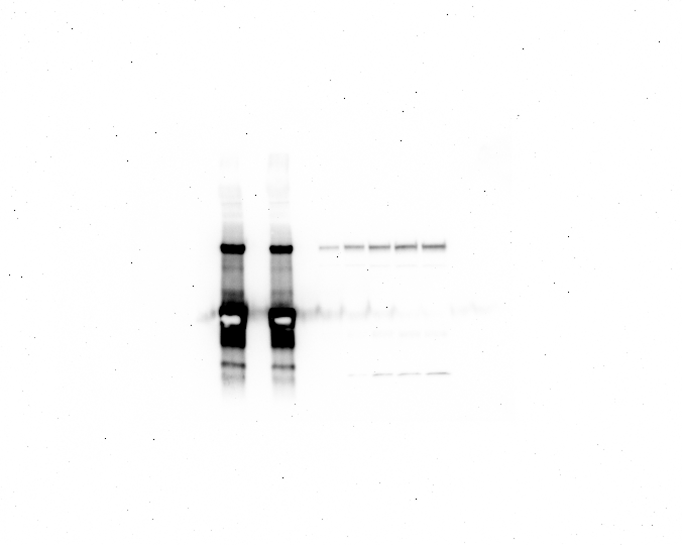


**Submitted Figure 9**


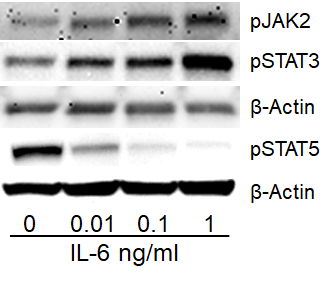


**Supplementary Gel blot for Figure 9**


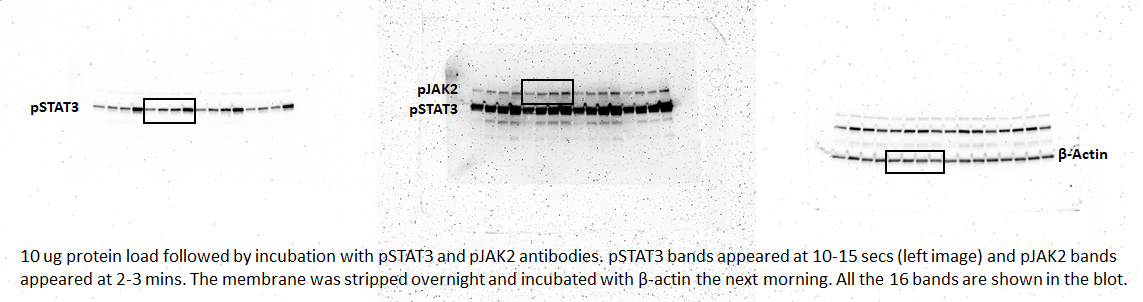


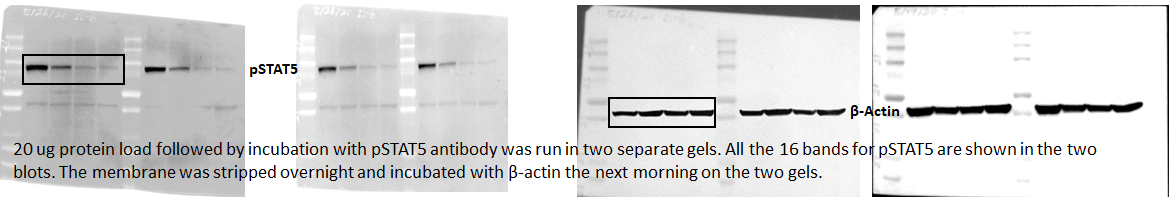

Supplement: Supplementary file 1 — Supplementary Information 1. [file 41598_2021_92751_MOESM1_ESM.docx]
